# Dasatinib regulates LPS-induced microglial and astrocytic neuroinflammatory responses by inhibiting AKT/STAT3 signaling

**DOI:** 10.1186/s12974-019-1561-x

**Published:** 2019-10-26

**Authors:** Ka-Young Ryu, Hyun-ju Lee, Hanwoong Woo, Ri-Jin Kang, Kyung-Min Han, HyunHee Park, Sang Min Lee, Ju-Young Lee, Yoo Joo Jeong, Hyun-Wook Nam, Youngpyo Nam, Hyang-Sook Hoe

**Affiliations:** 1 Department of Neural Development and Disease, Korea Brain Research Institute (KBRI), 61, Cheomdan-ro, Dong-gu, Daegu, 41068 South Korea; 2 Department of Brain & Cognitive Sciences, Daegu Gyeongbuk Institute of Science & Technology (DGIST), 333 Techno Jungang-daero, Hyeonpung-myeon, Dalseong-gun, Daegu, 42988 South Korea

**Keywords:** LPS, Neuroinflammation, STAT3, AKT, Microglia, Astrocytes

## Abstract

**Background:**

The FDA-approved small-molecule drug dasatinib is currently used as a treatment for chronic myeloid leukemia (CML). However, the effects of dasatinib on microglial and/or astrocytic neuroinflammatory responses and its mechanism of action have not been studied in detail.

**Methods:**

BV2 microglial cells, primary astrocytes, or primary microglial cells were treated with dasatinib (100 or 250 nM) or vehicle (1% DMSO) for 30 min or 2 h followed by lipopolysaccharide (LPS; 200 ng/ml or 1 μg/ml) or PBS for 5.5 h. RT-PCR, real-time PCR; immunocytochemistry; subcellular fractionation; and immunohistochemistry were subsequently conducted to determine the effects of dasatinib on LPS-induced neuroinflammation. In addition, wild-type mice were injected with dasatinib (20 mg/kg, intraperitoneally (i.p.) daily for 4 days or 20 mg/kg, orally administered (p.o.) daily for 4 days or 2 weeks) or vehicle (4% DMSO + 30% polyethylene glycol (PEG) + 5% Tween 80), followed by injection with LPS (10 mg/kg, i.p.) or PBS. Then, immunohistochemistry was performed, and plasma IL-6, IL-1β, and TNF-α levels were analyzed by ELISA.

**Results:**

Dasatinib regulates LPS-induced proinflammatory cytokine and anti-inflammatory cytokine levels in BV2 microglial cells, primary microglial cells, and primary astrocytes. In BV2 microglial cells, dasatinib regulates LPS-induced proinflammatory cytokine levels by regulating TLR4/AKT and/or TLR4/ERK signaling. In addition, intraperitoneal injection and oral administration of dasatinib suppress LPS-induced microglial/astrocyte activation, proinflammatory cytokine levels (including brain and plasma levels), and neutrophil rolling in the brains of wild-type mice.

**Conclusions:**

Our results suggest that dasatinib modulates LPS-induced microglial and astrocytic activation, proinflammatory cytokine levels, and neutrophil rolling in the brain.

**Electronic supplementary material:**

The online version of this article (10.1186/s12974-019-1561-x) contains supplementary material, which is available to authorized users.

## Background

The human brain comprises numerous cell types, including microglia, astrocytes, and neuronal cells. Among these cell types, microglia and astrocytes play important roles in physiological function in the normal brain (e.g., neuroprotection, phagocytosis) [1]. In the central nervous system (CNS), the physiological roles of microglia include synaptic pruning and neuronal plasticity [2]. Abnormal activation or functional impairment of microglia/astrocytes in the brain increases neuroinflammatory responses, leading to neurodegenerative diseases, including Alzheimer’s disease (AD) [3]. Increased neuroinflammatory responses can result in the release of various proinflammatory cytokines and mediators (i.e., IL-1β, COX-2, TNF-α), which affect hippocampus volume and cognitive/synaptic function [4]. Therefore, modulation of the neuroinflammatory response and microglial/astrocytic function in the brain is a potentially useful therapeutic strategy for neuroinflammation- and neurodegeneration-related diseases.

Dasatinib is a multi-target oral small-molecule inhibitor of tyrosine kinases (including BCR-ABL and Src family kinases) that has immunomodulating properties and can cross the blood-brain barrier (BBB) [5]. As an FDA-approved drug, dasatinib is currently used as a treatment for imatinib-resistant chronic myeloid leukemia (CML) [6–8]. Dasatinib can also regulate tumor cell proliferation, invasion, and metastasis in breast and glioma cells [9]. Recent studies have shown that dasatinib can decrease lung inflammatory responses in an acute silicosis model and in asthmatic lungs [10, 11]. In mice, Fraser et al. demonstrated that orally administered dasatinib significantly downregulates LPS-induced serum levels of TNF-α but not other proinflammatory cytokines in the lung [12]. In addition, Futosi et al. found that dasatinib can regulate proinflammatory responses in mature human neutrophils [13]. Moreover, dasatinib suppresses Aβ-induced TNF-α levels in primary microglial cultures, and dasatinib-injected APP/PS1-overexpressing mice (a mouse model of AD) exhibit significantly reduced microglial activation [14]. However, few studies have examined whether dasatinib can alter LPS-induced neuroinflammatory responses in vitro and in vivo and its molecular mechanism of action.

Here, we tested the effect of dasatinib on LPS-induced neuroinflammatory responses and found that dasatinib altered LPS-induced proinflammatory cytokine and anti-inflammatory cytokine levels in BV2 microglial cells, primary microglial cells, and primary astrocytes. In BV2 microglial cells, dasatinib inhibited TLR4/AKT and/or TLR4/ERK signaling to modulate LPS-stimulated neuroinflammatory responses. In addition, intraperitoneal and oral administration of dasatinib in wild-type mice resulted in decreases in microglial/astrocyte activation, LPS-induced proinflammatory cytokine levels (in the brain and plasma), and neutrophil rolling in the brain. Taken together, our data suggest that dasatinib can affect LPS-induced neuroinflammation in BV2 microglial cells, primary microglial cells, primary astrocytes, and wild-type mice.

## Methods

### Cell lines and culture conditions

To measure the effects of dasatinib on LPS-induced neuroinflammation, we used BV2 microglial cells (a generous gift from Dr. Kyung-Ho Suk). BV2 microglial cells were maintained in high-glucose DMEM (Invitrogen, Carlsbad, CA, USA) with 5% fetal bovine serum (FBS, Invitrogen, Carlsbad, CA, USA) in a 5% CO_2_ incubator.

### Rat primary astrocyte cultures

To examine whether dasatinib alters LPS-induced proinflammatory cytokine levels under high-glucose conditions, rat primary mixed glial cell cultures were prepared from 1-day-old Sprague Dawley rats. Briefly, rat cortices were triturated into single cells in high-glucose DMEM containing 10% FBS/penicillin-streptomycin solution (5000 units/mL penicillin, 5 mg/mL streptomycin, 4500 mg/L glucose, Corning, Mediatech Inc., Manassas, VA, USA) and plated onto 75 T flasks for 2 weeks. The 75 T flasks were then shaken at 120 rpm for 2 h to facilitate microglial detachment from the 75 T flask. The conditioned medium was removed, and the cells were washed with PBS three times, trypsinized, and centrifuged at 2000 rpm for 30 min. After centrifugation, the pellet was resuspended in high-glucose DMEM containing 10% FBS/penicillin-streptomycin and plated in 12-well plates to conduct experiments.

### Mouse primary astrocyte cultures

To determine the effects of dasatinib on LPS-induced neuroinflammatory responses under high- and low-glucose conditions, mouse mixed glial cultures were prepared from 1-day-old C57BL/N mice. Briefly, whole brains of postnatal 1-day-old C57BL/6N mice were chopped and mechanically minced using a 70-μm nylon mesh. The mixed cells were seeded in 75 T flasks and grown in low- or high-glucose DMEM (1000 mg/L glucose or 4500 mg/L glucose, respectively) supplemented with 10% FBS, 100 unit/mL penicillin, and 100 μg/mL streptomycin. Mixed glial cultures (primary astrocytes and primary microglial cells) were maintained in a 5% CO_2_ incubator at 37 °C for 2 weeks. To obtain mouse primary astrocytes, 75 T flasks containing mixed glial cells were sealed with foil and shaken at 250 rpm on a rotary shaker at RT overnight. The conditioned culture medium was then discarded, and the cells were dissociated using trypsin-EDTA and centrifuged at 2000 rpm for 30 min. After centrifugation, the pellet (primary astrocytes) was collected and used for experiments.

### Mouse primary microglial cultures

To investigate the effect of dasatinib on LPS-evoked neuroinflammatory responses under high- and low-glucose conditions, mouse mixed glial cultures were prepared from 1-day-old C57BL/N mice. Briefly, whole brains of postnatal 1-day-old C57BL/6N mice were minced using a 70-μm nylon mesh. The mixed cells were seeded in 75 T flasks and grown in low- or high-glucose DMEM (1000 mg/L glucose or 4500 mg/L glucose, respectively) supplemented with 10% FBS, 100 unit/mL penicillin, and 100 μg/mL streptomycin. Mixed glial cultures were maintained in a 5% CO_2_ incubator at 37 °C for 3–4 weeks. Mixed glial cultures were incubated with 0.25% trypsin diluted 1:4 in serum-free DMEM for 20–30 min in a 5% CO_2_ incubator at 37 °C. The upper layer of astrocytes was discarded, and the remaining microglia were used for analysis.

### Wild-type mice

To examine whether dasatinib affects neuroinflammatory responses in vivo, we used wild-type mice. All in vivo experiments were performed in accordance with approved animal protocols and guidelines established by the Korea Brain Research Institute (IACUC-2016-0013). Male C57BL6/N mice (8 weeks old, 25–30 g) were purchased from Orient-Bio Company (Gyeonggi-do, Korea) and housed in a pathogen-free facility with 12 h of light and dark per day at an ambient temperature of 22 °C. Wild-type mice were intraperitoneally (i.p.) injected with dasatinib (20 mg/kg) or vehicle (4% DMSO + 30% PEG + 5% Tween 80) daily for 4 days and injected with LPS (Sigma, *Escherichia coli,* 10 mg/kg, i.p.) or PBS. In addition, wild-type mice were orally administered dasatinib (20 mg/kg, p.o.) or vehicle (4% DMSO + 30% PEG + 5% Tween 80) daily for 4 days or daily for 2 weeks and injected with LPS (Sigma, *Escherichia coli*, 10 mg/kg, i.p.) or PBS. Three hours after LPS or PBS injection, the mice were perfused and fixed, and immunohistochemistry was performed. All data were analyzed in a semi-automated manner using Image J software, and all in vivo results were confirmed by an independent researcher who did not participate in the current experiments.

### Immunohistochemistry

To test the effects of dasatinib on LPS-induced microglial/astrocyte activation, proinflammatory cytokine levels, and neutrophil rolling, wild-type mice were administered dasatinib (20 mg/kg, i.p. daily for 4 days or 20 mg/kg, p.o. daily for 4 days or 2 weeks) or vehicle (4% DMSO + 30% PEG + 5% Tween 80) and injected with LPS (Sigma, *Escherichia coli,* 10 mg/kg, i.p.) or PBS. Three hours after LPS or PBS injection, the mice were perfused and fixed with 4% paraformaldehyde (PFA) solution, and mouse brain tissues were flash-frozen and sliced using a cryostat (35 μm thickness). Each brain section was rinsed with PBS three times and permeabilized with PBS containing 0.2% Triton X-100 and 1% BSA for 1 h at room temperature. The brain sections were then washed twice with 1% BSA and incubated with primary anti-Iba-1, anti-GFAP, anti-COX-2, anti-IL-6, anti-Ly-6B (neutrophil marker), or anti-ICAM-1 (endothelial cell marker) antibodies at 4 °C overnight. The next day, the brain sections were washed three times with PBS and incubated with Alexa 555-conjugated anti-rabbit IgG (1:200, Life Technologies), anti-goat IgG (1:200, Life Technologies), or anti-rat IgG (1:200, Abcam) for 1 h 30 min at room temperature. The brain sections were then rinsed three times with PBS, mounted on a glass slide, and covered with DAPI-containing mounting solution (Vector Laboratories). Images were acquired by a fluorescence microscope at × 5 or × 10 (DMi8, Leica Microsystems, Wetzlar, Germany). For this study, we used 8–9 male wild-type mice per group, and 2–3 slices of each brain from − 1.70 to − 2.06 mm relative to the bregma in stereotaxic coordinates were used to quantify the fluorescence intensity of anti-Iba-1, anti-GFAP,anti-COX-2, and anti-IL-6 in the cortex and hippocampus (*n* = 8–9 mice/group). To quantify the levels of anti-Iba-1, anti-GFAP, anti-COX-2, and anti-IL-6 in the cortex and hippocampus, the area of each region was measured by drawing a region of interest (ROI) in a DAPI fluorescence image using Image J software (NIH). The selected ROIs were overlaid on matching red fluorescence images, and the fluorescence intensity inside the overlaid ROIs was measured. The levels of anti-Iba-1, anti-GFAP, anti-COX-2, and anti-IL-6 were quantified as the measurement of the fluorescence intensity divided by the selected ROI area [15].

### Antibodies and inhibitors

To determine whether dasatinib affects LPS-induced neuroinflammation in vitro and in vivo, we used the following primary antibodies for western blotting (WB), immunocytochemistry (ICC), and immunohistochemistry (IHC): rabbit anti-COX-2 (1:200 for ICC, Abcam), rabbit anti-IL-1β (1:200 for ICC, Abcam), rat anti-mouse CD11b (1:400 for ICC, Abcam), rabbit anti-TLR4 (1:1000 for WB, Thermo Scientific, Waltham, MA, USA), rabbit anti-TLR4 (1:1000 for WB, Novus Biologicals, Littleton, CO, USA), rabbit anti-AKT (1:1000 for WB, Santa Cruz Biotechnology), rabbit anti-p-AKT (Ser473) (1:1000 for WB, Cell Signaling Technology), rabbit anti-ERK (1:1000 for WB, Santa Cruz Biotechnology), rabbit anti-p-ERK (Thr42/44) (1:1000 for WB, Cell Signaling Technology), rabbit anti-STAT3 (1:1000 for WB, Cell Signaling Technology), rabbit anti-p-STAT3 (Ser727, 1:1000 for WB, 1:200 for ICC, Abcam), rabbit anti-P38 (1:1000 for WB, Cell Signaling Technology), rabbit anti-p-P38 (1:1000 for WB, Cell Signaling Technology), rabbit anti-GFAP (1:500 for IHC, Wako), rabbit anti-Iba-1 (1:500 for IHC, Wako), rabbit anti-COX-2 (1:500 for IHC, Abcam), goat anti-IL-6 (1:50 for IHC, Santa Cruz Biotechnology), rat anti-Ly-6B (1:500 for IHC, Bio-Rad), and mouse anti-ICAM-1 (1:200 for IHC, Santa Cruz Biotechnology).

To examine whether dasatinib affects TLR4/AKT/ERK/STAT3 signaling to modulate the LPS-induced neuroinflammatory response, we used a TLR4 inhibitor (TAK-242, 500 nM, Calbiochem), AKT inhibitor (MK2206, 10 μM, Selleckchem), ERK inhibitor (PD98059, 10 μM, Calbiochem), and STAT3 inhibitor (S3I-201, 50 μM, Sigma-Aldrich) in our experiments. LPS from *Escherichia coli* O111:B4 was purchased from Sigma-Aldrich (St. Louis, MO, USA).

### Cell viability assays

#### MTT assay

To determine the effects of dasatinib on cytotoxicity in BV2 microglial cells and mouse primary astrocytes, cell viability was assessed using the 3-(4,5-dimethylthiazol-2-yl)-2,5-diphenyltetrazolium bromide (MTT) assay. BV2 microglial cells and mouse primary astrocytes were separately seeded in 96-well plates (4 × 10^4^ cells/well) and treated with various concentrations of dasatinib (100, 250, 500, 750, 1000 nM) for 24 h. The cells were then treated with 0.5 mg/ml MTT and incubated in a 5% CO_2_ incubator at 37 °C for 3 h, and the absorbance was measured at 570 nm. In addition, to test the cytotoxic effects of dasatinib on BV2 or mouse primary astrocytes in the presence of LPS, BV2 microglial cells and mouse primary astrocytes were separately seeded in 96-well plates and treated with dasatinib (250 nM) or vehicle for 30 min followed by LPS (200 ng/ml) or PBS for 23.5 h. The cells were then treated with 0.5 mg/ml MTT and incubated in a 5% CO_2_ incubator at 37 °C for 3 h, and the absorbance was measured at 570 nm.

#### BrdU assay

To investigate the effects of dasatinib on the proliferation of BV2 microglial cells via a non-metabolic assay, a BrdU assay kit (Cell Signaling, Danvers, MA, USA) was used. BV2 microglial cells were seeded in 96-well plates at a density of 4 × 10^4^ cells/well and treated with various concentrations of dasatinib (100, 250, 500, 750, and 1000 nM) for 24 h in the absence of FBS. In addition, BV2 microglial cells were seeded in 96-well plates and treated with dasatinib (250 nM) or vehicle (1% DMSO) for 30 min followed by LPS (200 ng/ml) or PBS for 23.5 h. The BrdU assays were conducted in accordance with the manufacturer’s instructions. The labeling time with BrdU (10 μM) was 4 h, and BrdU incorporation was detected by an anti-BrdU antibody and HRP-conjugated secondary antibody. The absorbance was measured at 450 nm. In addition, we conducted parallel experiments with mouse primary astrocytes in the presence/absence of LPS treatment using the BrdU assay.

### Reverse transcription-polymerase chain reaction (RT-PCR)

To determine whether dasatinib modulates LPS-induced proinflammatory cytokine levels, BV2 microglial cells (2.5 × 10^5^ cells/well) and mouse/rat primary astrocytes (7.0 × 10^5^ cells/well) were treated with dasatinib (100 nM or 250 nM) or vehicle (1% DMSO) for 30 min followed by LPS (200 ng/ml or 1 μg/ml) or PBS for 5 h 30 min, and total RNA was extracted using TRIzol (Invitrogen) according to the manufacturer’s instructions. Total RNA was reverse transcribed to cDNA using a Superscript cDNA Premix Kit II with oligo (dT) primers (GeNetBio, Korea), and RT-PCR was performed using Prime Taq Premix (GeNetBio, Korea). To examine the effects of dasatinib on the LPS-induced neuroinflammatory response in BV2 microglial cells and mouse primary astrocytes, we used the following primers: IL-1β: Forward (F)′, AGC TGG AGA GTG TGG ATC CC, and Reverse (R)′, CCT GTC TTG GCC GAG GAC TA; IL-6: F′, CCA CTT CAC AAG TCG GAG GC, and R′, GGA GAG CAT TGG AAA TTG GGG T; COX-2: F′, GCC AGC AAA GCC TAG AGC AA, and R′, GCC TTC TGC AGT CCA GGT TC; iNOS: F′, CCG GCA AAC CCA AGG TCT AC, and R′, GCA TTT CGC TGT CTC CCC AA; TNF-α: F′, CTA TGG CCC AGA CCC TCA CA, and R′, TCT TGA CGG CAG AGA GGA GG; GAPDH: F′, CAG GAG CGA GAC CCC ACT AA, and R′, ATC ACG CCA CAG CTT TCC AG. For rat primary astrocytes, we used the following primers for RT-PCR: COX-2: F′, TCC AAC TCA AGT TCG ACC CA, and R′, TCC TCC GAA GGT GCT AGG TT; IL-1β: F′, AAA ATG CCT CGT GCT GTC TG, and R′, CAG AAT GTG CCA CGG TTT TC; IL-6: F′, TTG CCT TCT TGG GAC TGA TG, and R′, TGG AAG TTG GGG TAG GAA GG; iNOS: F′, ATC ATG GAC CAC CAC ACA GC, and R′, GGT GTT GAA GGC GTA GCT GA; TNF-α: F′, AGC ACA GAA AGC ATG ATC CG, and R′, CTC CCT CAG GGG TGT CCT TA; GAPDH: F′, GTT ACC AGG GCT GCC TTC TC, and R′, GTG ATG GCA TGG ACT GTG GT. The RT-PCR products were separated by electrophoresis on 1.8% agarose gels and photographed, and the images were analyzed using ImageJ (NIH) and Fusion software (Korea).

### Real-time quantitative PCR (qPCR)

To examine the effects of dasatinib on LPS-induced proinflammatory and anti-inflammatory cytokine levels, BV2 microglial cells (2.5 × 10^5^ cells/well) and mouse primary astrocytes (7.0 × 10^5^ cells/well) were treated with dasatinib (250 nM) or vehicle (1% DMSO) for 30 min followed by LPS (200 ng/ml or 1 μg/ml) or PBS treatment for 5 h 30 min, and total RNA was extracted using TRIzol (Invitrogen) according to the manufacturer’s instructions. In addition, to test whether dasatinib alters LPS-induced anti-inflammatory cytokine levels, mouse primary microglial cells (7.0 × 10^5^ cells/well) were treated with dasatinib (250 nM) or vehicle (1% DMSO) for 2 h followed by LPS (200 ng/ml) or PBS treatment for 5 h 30 min, and total RNA was extracted using TRIzol (Invitrogen) according to the manufacturer’s instructions. Total RNA was reverse transcribed to cDNA using a Superscript cDNA Premix Kit II with oligo (dT) primers (GeNetBio, Korea), and real-time PCR was performed using Fast SYBR Green Master Mix (Thermo Fisher Scientific, CA, USA). To assess the effects of dasatinib on the LPS-induced neuroinflammatory response in BV2 microglial cells, mouse primary astrocytes and mouse primary microglia, we used the following primers: COX-2: Forward (F)′, CCA CTT CAA GGG AGT CTG GA, and Reverse (R)′, AGT CAT CTG CTA CGG GAG GA; IL-6: F′, CCA CGG CCT TCC CTA CTT C, and R′, TTG GGA GTG GTA TCC TCT GTG A; iNOS: F′, GGA TCT TCC CAG GCA ACC A, and R′, TCC ACA ACT CGC TCC AAG ATT; TNF-α: F′, TCC AGG CGG TGC CTA TGT, and R′, GCC CCT GCC ACA AGC A; IL-1β: F′, TTG ACG GAC CCC AAA AGA TG, and R′, AGG ACA GCC CAG GTC AAA G; MRC-1: F′, CCC AAG GGC TCT TCT AAA GCA, and R′, CGC CGG CAC CTA TCA CA; IL-10: F′, GAT GCC CCA GGC AGA GAA, and R′, CAC CCA GGG AAT TCA AAT GC; IL-4: F′, CCC ACC TGC TTC TCT GAC TAC A, and R′, CAG CGC TAT CCA GGA ACC A; GAPDH: F′, TGG GCT ACA CTG AGG ACC ACT, and R′, GGG AGT GTC TGT TGA AGT CG. The samples were amplified and quantified on a QuantStudio 5 Real-Time PCR System (Thermo Fisher Scientific, San Jose, CA, USA). The cycle threshold (Ct) values of the mRNA of inflammatory factors were normalized to the Ct value for *Gapdh*. The data were quantified as the fold change relative to the vehicle-treated control.

### Enzyme-linked immunosorbent assay (ELISA)

To assess whether dasatinib affects circulating proinflammatory cytokine levels, wild-type mice were injected with dasatinib (20 mg/kg, i.p. daily for 4 days or 20 mg/kg, p.o. daily for 4 days or 2 weeks) or vehicle (4% DMSO + 30% PEG + 5% Tween 80) followed by injection with LPS (Sigma, *Escherichia coli*, 10 mg/kg, i.p.) or PBS. Three hours after LPS or PBS injection, blood was collected in EDTA-coated tubes (Becton, Dickinson, and Company, Franklin Lakes, NJ, USA), and plasma was isolated by centrifugation at 2000 g for 10 min at 4 °C. Plasma IL-6, IL-1β, and TNF-α levels were measured using Duoset ELISA kits (Cat. no. DY406, DY401, and DY410, respectively, R&D Systems, Minneapolis, MN, USA) in accordance with the manufacturer’s instructions.

### Immunocytochemistry

To examine the effect of dasatinib on LPS-induced neuroinflammatory responses, BV2 microglial cells and mouse primary astrocytes were fixed with 4% paraformaldehyde for 10 min, washed three times with 1x PBS, and then incubated with anti-CD11b and anti-COX-2, anti-CD11b and anti-p-AKT, anti-CD11b and anti-p-ERK, or anti-p-STAT3 antibodies in GDB buffer (0.1% gelatin, 0.3% Triton X-100, 16 mM sodium phosphate, pH 7.4, and 450 mM NaCl) overnight at 4 °C. The next day, BV2 microglial cells and mouse primary astrocytes were washed three times with 1x PBS and incubated with Alexa Fluor 488-conjugated anti-mouse and Alexa Fluor 555-conjugated anti-rabbit antibodies (1:200, Molecular Probes, USA) for 1 h at room temperature. The cells were mounted in DAPI-containing solution (Vector Laboratories, CA, USA), and images were captured from a single plane using a confocal microscope (Nikon, Japan) and analyzed using ImageJ software. Samples were analyzed in a blinded manner using 5–10 individual images.

### Western blotting

To examine whether dasatinib regulates AKT and ERK signaling, BV2 microglial cells (2.5 × 10^5^ cells/well), mouse primary microglial cells (7.0 × 10^5^ cells/well), and mouse primary astrocytes (7.0 × 10^5^ cells/well) were treated with dasatinib (250 nM) or vehicle (1% DMSO) for 45 min, followed by LPS (1 μg/ml) or PBS treatment for 45 min. The cells were lysed in RIPA buffer containing protease and phosphatase inhibitor tablets (Roche, USA), and western blot analyses were performed as previously described [16]. Images were analyzed using Fusion software or Image J software.

### Subcellular fractionation (cytosol vs nucleus)

To test whether dasatinib affects LPS-induced cytosolic and nuclear p-STAT3 levels, BV2 microglial cells were treated with dasatinib (250 nΜ) or vehicle (1% DMSO) for 30 min followed by LPS (1 μg/ml) or PBS for 5.5 h. The cells were then lysed in cytosol fractionation buffer (10 mM HEPES, pH 8.0, 1.5 mM MgCl_2_, 10 mM KCl, 0.5 mM DTT, 300 mM sucrose, 0.1% NP-40, and 0.5 mM PMSF) for 5 min. The cell lysates were centrifuged at 10,000 rpm for 1 min at 4 °C, and the supernatant was stored as the cytosolic fraction. Nuclear fractionation buffer (10 mM HEPES, pH 8.0, 20% glycerol, 100 mM KCl, 100 mM NaCl, 0.2 mM EDTA, 0.5 mM DTT, and 0.5 mM PMSF) was added to the pellet, followed by lysis on ice for 15 min. The sample was then centrifuged at 10,000 rpm for 15 min at 4 °C, and western blotting was conducted with anti-p-STAT3, β-actin, or PCNA antibodies. Data were analyzed using Fusion or Image J software.

### Cell surface biotinylation

To determine if dasatinib alters cell surface level of TLR4, BV2 microglial cells were treated with dasatinib (250 nM) or vehicle (1% DMSO) for 30 min followed by LPS (1 μg/ml) or PBS for 5.5 h. Surface proteins were then labeled with EZ-Link Sulfo-NHS-SS-Biotin (Thermo Fisher Scientific, Pittsburgh, PA, USA) under gentle shaking at 4 °C for 30 min (Thermo Fisher Scientific, Cat No: 1859389). After 30 min, 50 μl of quenching solution was added to stop the reaction, and the surface-labeled cells were lysed in lysis buffer, disrupted by sonication on ice, and incubated on ice for 30 min. The cells were then centrifuged at 10,000 rpm for 10 min, and the lysate was added to immobilized NeutroAvidin TM gel beads and incubated for 1 h at room temperature. To elute the biotinylated protein, the beads were washed with lysis buffer three times and incubated with elution buffer (80 mM DTT in SDS-PAGE sample buffer, Bio-Rad, 161–0747) for 1 h at RT, heated at 95 °C for 5 min, and centrifuged at 10,000 rpm for 5 min. Surface proteins were then analyzed by western blotting with an antibody recognizing the N-terminus of TLR4.

### Griess assay (NO assay)

To determine the effects of dasatinib on nitrite (NO) production, we conducted the Griess assay as previously described [17]. Briefly, BV2 microglial cells (2.5 × 10^5^ cells/well) were treated with dasatinib (250 nM) or vehicle (1% DMSO) for 30 min followed by LPS (100 ng/mL) or PBS treatment for 23.5 h. The conditioned medium was mixed with Griess reagent (including 0.1% *N*-(1-naphthyl) ethylenediamine dihydrochloride and 1% sulfanilamide in 2% phosphoric acid) in 96-well plates and incubated at room temperature for 5 min. The absorbance was measured at 540 nm, and the levels of nitrite (NO) were analyzed by reference to a standard curve of sodium nitrite.

### Statistical analyses

All data were analyzed with GraphPad Prism 6 software using either unpaired two-tailed *t* tests with Welch’s correction for comparisons between two groups or one-way ANOVA for multiple comparisons. Post hoc analyses were performed with Tukey’s multiple-comparison test with significance set at *p < 0.05, **p < 0.01, and ***p < 0.001. Data are presented as the mean ± S.E.M.

## Results

### Dasatinib significantly decreases LPS-induced proinflammatory cytokine COX-2 and IL-6 mRNA levels in BV2 microglial cells

**Fig. 1:**
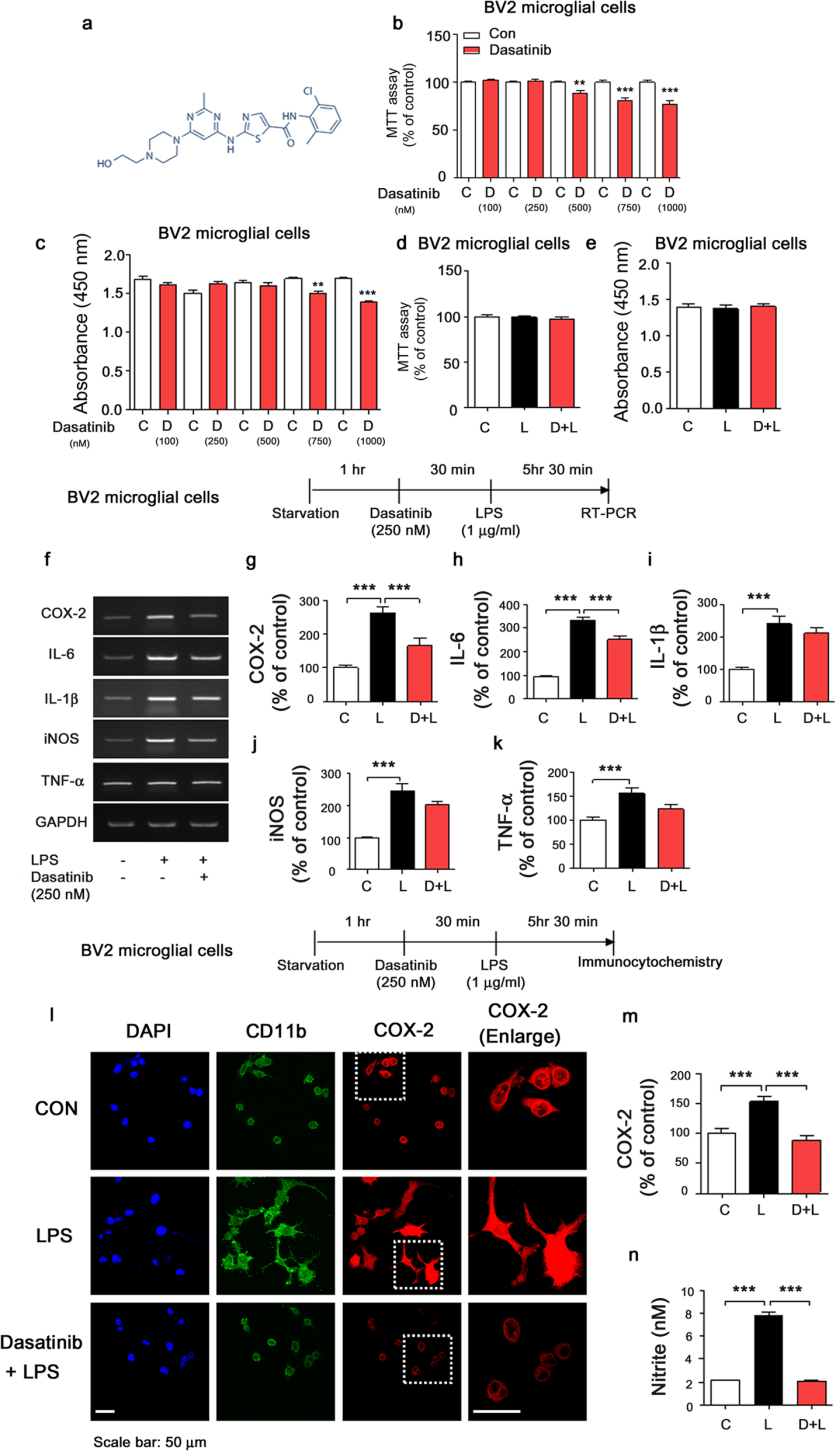
The effect of dasatinib on LPS-induced proinflammatory cytokine levels in BV2 microglial cells. **a** Structure of dasatinib. **b** BV2 microglial cells were treated with vehicle (1% DMSO) or dasatinib (100, 250, 500, 750, or 1000 nM) for 24 h, and MTT assays were conducted (*n* = 14 experimental replicates per dose). **c** BV2 microglial cells were treated with vehicle (1% DMSO) or dasatinib (100, 250, 500, 750, or 1000 nM) for 24 h, and BrdU proliferation assays were conducted (100 nM, *n* = 5; 250 nM, *n* = 5; 500 nM, *n* = 5; 750 nM, *n* = 5; or 1000 nM, *n* = 5). **d** BV2 microglial cells were treated with dasatinib (250 nM) or vehicle (1% DMSO) for 30 min followed by LPS (200 ng/ml) or PBS for 23 h 30 min, and MTT assays were performed (con, *n* = 6; LPS, *n* = 6; dasatinib+LPS, *n* = 6). **e** BV2 microglial cells were treated with dasatinib (250 nM) or vehicle (1% DMSO) for 30 min followed by LPS (200 ng/ml) or PBS for 23 h 30 min, and BrdU proliferation assays were performed (con, *n* = 6; LPS, *n* = 6; dasatinib+LPS, *n* = 6). **f**–**k** BV2 microglial cells were treated with dasatinib (250 nM) or vehicle (1% DMSO) for 30 min followed by LPS (1 μg/ml) or PBS for 5.5 h, and proinflammatory cytokine levels were measured by RT-PCR (COX2, IL-1β, and TNF-alpha: con, *n* = 21; LPS, *n* = 21; dasatinib+LPS, *n* = 21; IL-6 and iNOS: con, *n* = 44; LPS, *n* = 44; dasatinib+LPS, *n* = 44). **l**, **m** BV2 microglial cells were treated with dasatinib (250 nM) or vehicle (1% DMSO) for 30 min, followed by treatment with LPS (1 μg/ml) or PBS for 5.5 h and immunostaining with anti-COX2 and anti-CD11b antibodies (con, *n* = 32; LPS, *n* = 54; dasatinib+LPS, *n* = 34). **n** BV2 microglial cells were treated with vehicle (1% DMSO) or dasatinib (250 nM) for 30 min followed by LPS (100 ng/ml) or PBS for 23 h 30 min. Levels of nitric oxide (NO) were measured using the Griess assay (con, *n* = 20; LPS, *n* = 20; dasatinib+LPS, *n* = 20). Two-tailed *t* tests (**b**, **c**) and one-way ANOVA with Tukey’s post hoc test (**d**–**n**) were used to analyze significant differences. **p < 0.01, ***p < 0.001

Dasatinib is a useful drug for chronic myelogenous leukemia (CML), but its effects on LPS-induced neuroinflammation and its mechanism of action are not well-studied. Therefore, we initially measured the cell cytotoxicity and proliferation effects of dasatinib in BV2 microglial cells via an MTT assay and non-metabolic cell proliferation (BrdU) assay. For these experiments, BV2 microglial cells were treated with vehicle (1% DMSO) or dasatinib (100, 250, 500, 750, or 1000 nM) for 24 h, and the MTT assay or BrdU proliferation assay was performed. We found that dasatinib did not exhibit BV2 microglial cell toxicity up to 250 nM in the MTT assay (Fig. 1a–b). Treatment with 500 nM, 750 nM, or 1000 nM dasatinib resulted in cytotoxic effects of 11%, 19%, and 23%, respectively, in the MTT assays of BV2 microglial cells (Fig. 1b). In addition, up to 500 nM dasatinib did not suppress BV2 microglial cell proliferation compared to vehicle treatment (Fig. 1c). However, treatment with 750 nM and 1000 nM dasatinib significantly reduced BrdU incorporation in BV2 microglial cells (Fig. 1c). We then assessed whether dasatinib affects the cell viability and cell proliferation of BV2 microglial cells in the presence of LPS. BV2 microglial cells were treated with dasatinib (250 nM) or vehicle (1% DMSO) for 30 min followed by LPS (200 ng/ml) or PBS for 23 h 30 min, and MTT assays or BrdU incorporation assays were performed. Treatment with dasatinib and LPS did not alter cell viability or proliferation compared to vehicle or LPS treatment in BV2 microglial cells (Fig. 1d, e).

To investigate whether dasatinib affects LPS-induced proinflammatory responses,

BV2 microglial cells were treated with vehicle (1% DMSO) or dasatinib (100 nM) for 30 min followed by LPS (1 μg/ml) or PBS for 5.5 h, and proinflammatory cytokine levels were measured using RT-PCR. At 100 nM, dasatinib significantly decreased LPS-induced proinflammatory cytokine COX-2 and IL-6 mRNA levels but no other cytokine mRNA levels (Additional file 1: Figure S1a–f).

We then examined whether a higher concentration of dasatinib can further regulate LPS-induced neuroinflammation. BV2 microglial cells were treated with vehicle (1% DMSO) or dasatinib (250 nM) for 30 min followed by LPS (1 μg/ml) or PBS for 5.5 h, and proinflammatory cytokine levels were measured using RT-PCR. At 250 nM, dasatinib significantly decreased LPS-induced proinflammatory cytokine COX-2 and IL-6 mRNA levels. In addition, decreasing trends of LPS-induced proinflammatory cytokine iNOS and TNF-α mRNA levels were observed in response to 250 nM dasatinib (Fig. 1f–k).

As a complementary approach, BV2 microglial cells were treated with vehicle (1% DMSO) or dasatinib (250 nM) for 30 min followed by LPS (1 μg/ml) or PBS for 5.5 h, and immunocytochemistry was performed with anti-CD11b and anti-COX-2 antibodies (Fig. 1l, m). Consistent with our findings above, dasatinib (250 nM) significantly suppressed LPS-induced COX-2 levels in BV2 microglial cells (Fig. 1l, m).

To examine whether dasatinib can significantly alter NO production in a longer treatment, BV2 microglial cells were treated with vehicle (1% DMSO) or dasatinib (250 nM) for 30 min followed by LPS (100 ng/mL) or PBS for 23 h 30 min, and the Griess assay was conducted. We found that dasatinib significantly reduced LPS-induced NO production in the longer treatment (Fig. 1n).

Next, we investigated the effect of dasatinib on the neuroinflammatory response induced by a lower concentration of LPS (200 ng/ml). For these experiments, BV2 microglial cells were treated with vehicle (1% DMSO) or dasatinib (250 nM) for 30 min followed by LPS (200 ng/ml) or PBS for 5.5 h, and proinflammatory cytokine levels were measured using RT-PCR. Dasatinib treatment markedly suppressed COX-2, IL-6, and TNF-α mRNA levels but not iNOS mRNA levels stimulated by 200 ng/ml LPS in BV2 microglial cells (Additional file 1: Figure S2a-e). Based on our findings in Fig. 1, dasatinib further suppressed LPS-induced proinflammatory cytokine levels in the low-dose LPS treatment compared to the high-dose LPS treatment (Fig. 1, Additional file 1: Figure S2a–d).

We further examined whether pretreatment with LPS followed by dasatinib treatment can modulate LPS-stimulated proinflammatory cytokine levels. BV2 microglial cells were treated with LPS (1 μg/ml) or PBS for 30 min followed by vehicle (1% DMSO) or dasatinib (250 nM) for 5.5 h, and proinflammatory cytokine levels were measured by RT-PCR. Surprisingly, we found that post-treatment with dasatinib significantly reduced only LPS-induced COX-2 mRNA levels and no other proinflammatory cytokine mRNA levels (Additional file 1: Figure S4a–f). Our findings suggest that pre-treatment with dasatinib is more effective than post-treatment in decreasing LPS-stimulated proinflammatory cytokine levels in BV2 microglial cells.

Finally, to test whether dasatinib modulates anti-inflammatory responses, BV2 microglial cells were treated with vehicle (1% DMSO) or dasatinib (250 nM) for 30 min followed by LPS (1 μg/ml) or PBS for 5 h 30 min, and anti-inflammatory mediator levels were measured by real-time PCR. We found that LPS alone did not significantly alter MRC-1 (M2 phenotype marker), IL-4, and IL-10 mRNA levels compared to vehicle treatment (Additional file 1: Figure S3a–c). Interestingly, dasatinib markedly increased anti-inflammatory cytokine IL-4 and IL-10 mRNA levels in LPS-treated BV2 microglial cells compared to LPS treatment (Additional file 1: Figure S3a–c). As a control experiment, dasatinib significantly suppressed LPS-induced proinflammatory cytokine IL-6 levels compared to LPS treatment (Additional file 1: Figure S3a–c). These data suggest that dasatinib can modulate anti-inflammatory responses in BV2 microglial cells.

### Dasatinib affects LPS-induced proinflammatory cytokine levels in mouse primary microglial cells

**Fig. 2:**
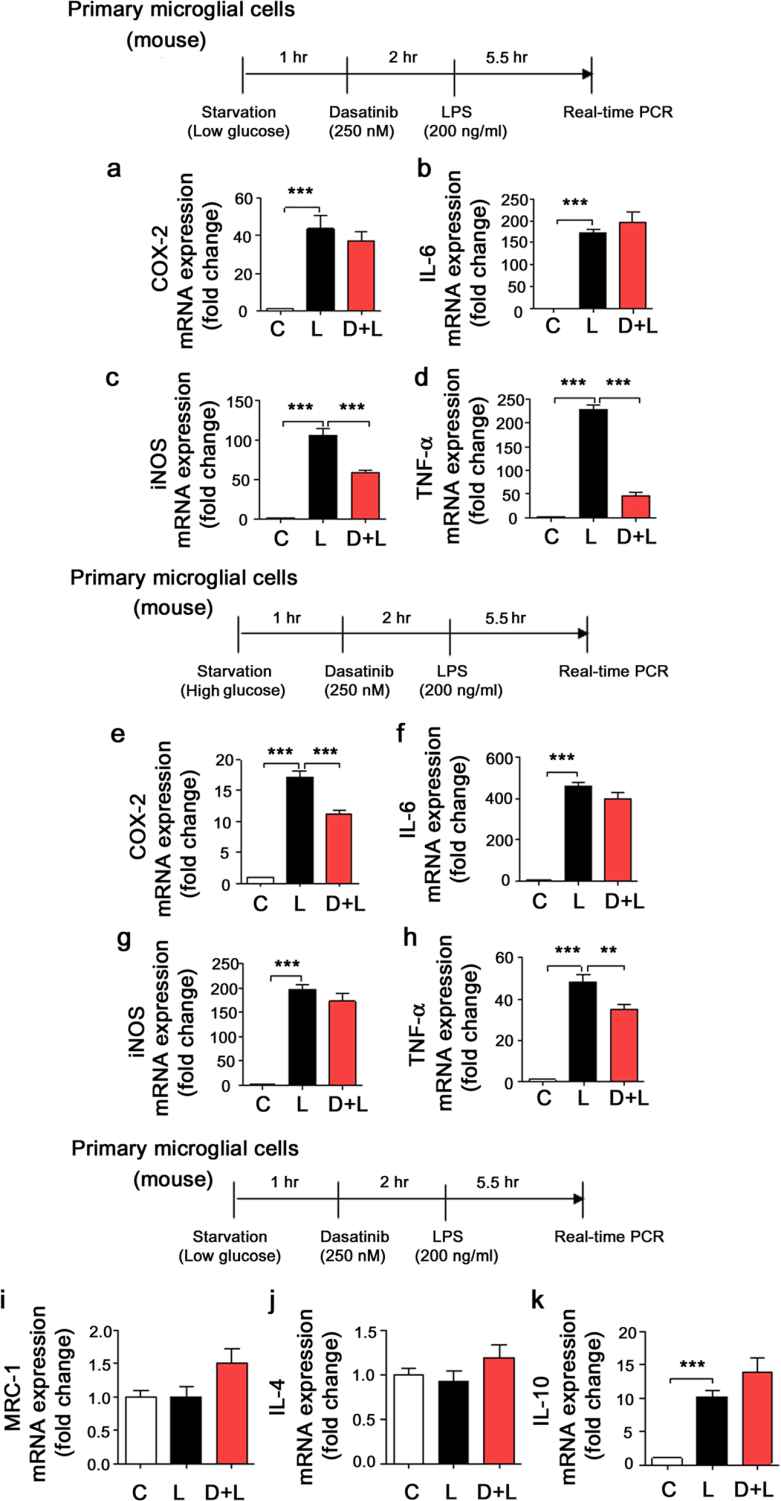
The effect of dasatinib on LPS-induced pro- and anti-inflammatory cytokine levels in primary microglial cells. **a**–**d** Primary microglial cells were treated with vehicle (1% DMSO) or dasatinib (250 nM) for 2 h followed by LPS (200 ng/ml) or PBS for 5.5 h under low-glucose conditions. Proinflammatory cytokine levels were then measured by real-time PCR (con, *n* = 6; LPS, *n* = 6; dasatinib+LPS, *n* = 6). **e**–**h** Primary microglia were treated with vehicle (1% DMSO) or dasatinib (250 nM) for 2 h followed by LPS (200 ng/ml) or PBS for 5.5 h under high-glucose conditions, and anti-proinflammatory cytokine levels were measured by real-time PCR (con, *n* = 6; LPS, *n* = 6; dasatinib+LPS, *n* = 6). **i**–**k** Primary microglia were treated with vehicle (1% DMSO) or dasatinib (250 nM) for 2 h followed by LPS (200 ng/ml) or PBS for 5.5 h under low-glucose conditions, and anti-inflammatory cytokine levels were measured by real-time PCR. (con, *n* = 6; LPS, *n* = 6; dasatinib+LPS, *n* = 6.) One-way ANOVA with Tukey’s post hoc test was used to analyze significant differences. ***p < 0.001

To determine whether dasatinib can alter LPS-induced neuroinflammation in primary microglial cells, we performed mouse primary microglial cell culture under low- and high-glucose conditions. For these experiments, mouse primary microglial cells were treated with dasatinib (250 nM) or vehicle for 2 h followed by LPS (200 ng/ml) or PBS for 5.5 h, and real-time PCR was performed. Interestingly, we found that dasatinib significantly reduced LPS-induced iNOS and TNF-α mRNA levels under low-glucose conditions, but not COX-2 and IL-6 mRNA levels (Fig. 2a–d). In addition, we observed that dasatinib treatment significantly inhibited LPS-stimulated COX-2 and TNF-α mRNA levels in primary microglial cells under high-glucose conditions (Fig. 2e–h), suggesting that dasatinib differentially regulates LPS-induced proinflammatory cytokine levels depending on the glucose level in mouse primary microglial cells.

To investigate the effects of dasatinib on anti-inflammatory cytokine levels, mouse primary microglial cells were treated with vehicle (1% DMSO) or dasatinib (250 nM) for 2 h followed by LPS (200 ng/ml) or PBS for 5.5 h, and anti-inflammatory mediator levels were measured by real-time PCR. We found that LPS treatment significantly increased anti-inflammatory IL-10 mRNA expression, whereas MRC-1 and IL-4 mRNA levels were not altered in mouse primary microglial cells under low-glucose conditions (Fig. 2i–k). Dasatinib-treated primary microglial cells exhibited a trend toward increased anti-inflammatory cytokine IL-4 and IL-10 mRNA levels in LPS-treated mouse primary microglial cells (Fig. 2i–k).

### Dasatinib suppresses TLR4 signaling to alter LPS-induced proinflammatory cytokine levels

**Fig. 3:**
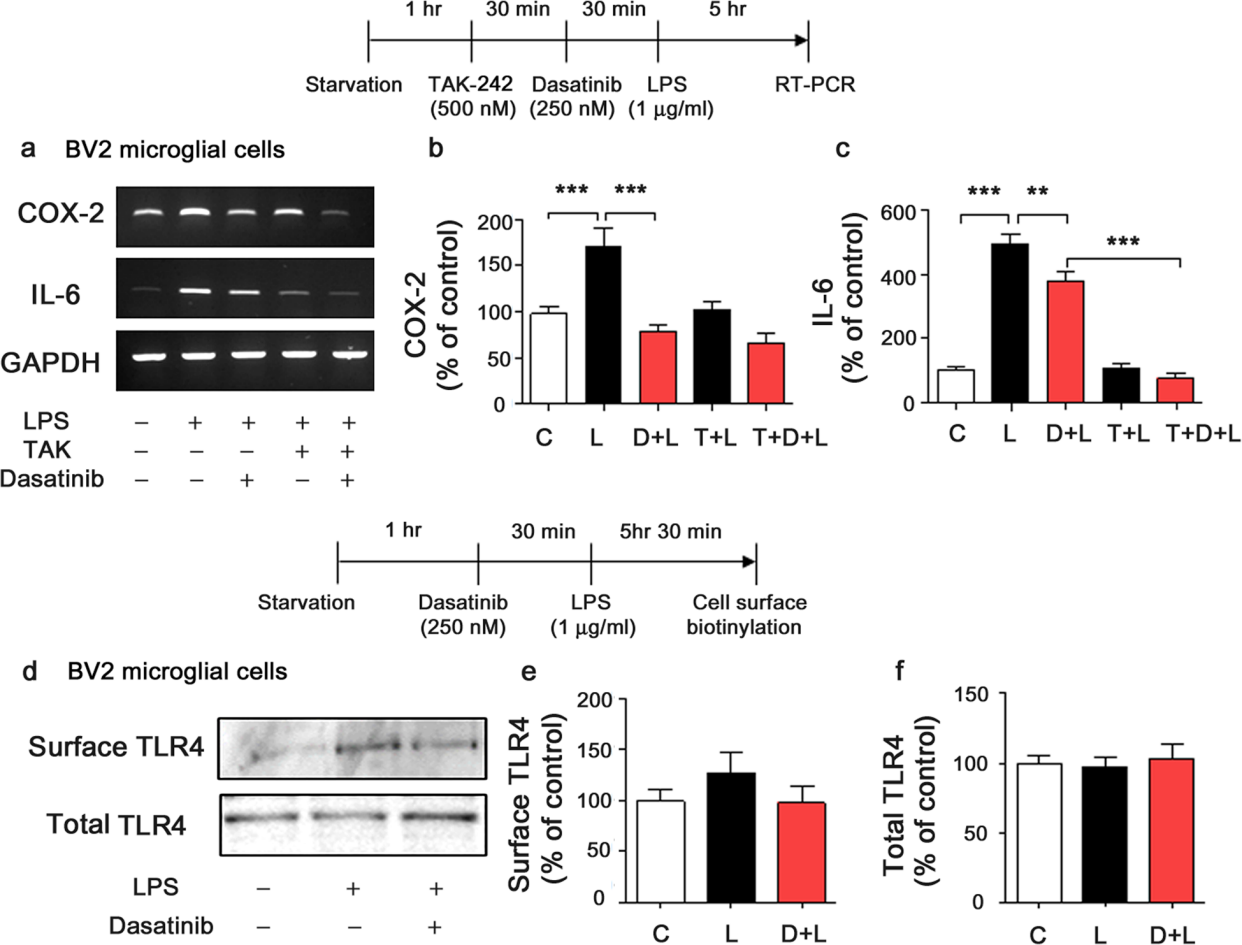
Dasatinib affects TLR4 signaling to regulate LPS-induced proinflammatory cytokine levels in BV2 microglial cells. **a** BV2 microglial cells were pretreated with TAK-242 (a TLR4 receptor inhibitor, 500 nM) or vehicle (1% DMSO) for 30 min, treated with dasatinib (250 nM) or vehicle (1% DMSO) for 30 min, and treated with LPS (1 μg/ml) or PBS for 5 h. The mRNA levels of COX-2 and IL-6 were analyzed by RT-PCR. **b**, **c** Quantification of the data in **a** (COX-2: con, *n* = 10; LPS, *n* = 10; dasatinib+LPS, *n* = 10; TAK-242+LPS, *n* = 10; TAK-242+dasatinib+LPS, *n* = 10; and IL-6: con, *n* = 14; LPS, *n* = 14; dasatinib+LPS, *n* = 14; TAK-242+LPS, *n* = 14; TAK-242+ dasatinib+LPS, *n* = 14). **d** BV2 microglial cells were treated with dasatinib (250 nM) or vehicle (1% DMSO) for 30 min followed by LPS (1 μg/ml) or PBS for 5.5 h, and cell surface biotinylation was conducted. **e**, **f** Quantification of the data in **d** (Surface TLR4: con, *n* = 11; LPS, *n* = 11; dasatinib+LPS, *n* = 11; Total TLR4: con, *n* = 16; LPS, *n* = 16; dasatinib+LPS, *n* = 16). One-way ANOVA with Tukey’s post hoc test was used to analyze significant differences. **p < 0.01, ***p < 0.001

To examine how dasatinib alters LPS-induced neuroinflammatory responses, we initially tested whether dasatinib inhibits TLR4 signaling to decrease LPS-induced neuroinflammation. For this experiment, we mainly tested COX-2 and IL-6 mRNA levels, since dasatinib only significantly reduced LPS-induced COX-2 and IL-6 mRNA levels in BV2 microglial cells (Fig. 1f–k). BV2 microglial cells were pre-treated with TAK-242 (TLR inhibitor, 500 nM) or vehicle (1% DMSO) for 30 min, treated with dasatinib (250 nM) or vehicle (1% DMSO) for 30 min, and subsequently treated with LPS (1 μg/ml) or PBS for 5 h. Consistent with our findings in Fig. 1, dasatinib significantly decreased LPS-induced COX-2 and IL-6 mRNA levels (Fig. 3a–c). In addition, treatment with TAK-242, dasatinib, and LPS (labeled as T+D+L) did not reduce LPS-induced COX-2 and IL-6 mRNA levels compared with treatment with LPS and TAK-242 (labeled as T+L) (Fig. 3a–c). These data indicate that dasatinib inhibits LPS-induced COX-2 and IL-6 mRNA levels by suppressing TLR-4 signaling.

Next, we investigated whether dasatinib regulates LPS-stimulated proinflammatory cytokine levels by preventing the interaction of LPS and TLR4. BV2 microglial cells were treated with dasatinib (250 nM) or vehicle (1% DMSO) for 30 min followed by LPS (1 μg/ml) or PBS for 5.5 h, and cell-surface biotinylation assays were conducted. LPS treatment increased cell-surface levels of TLR4 compared to the control treatment (Fig. 3d, e). A trend toward decreased LPS-induced cell-surface levels of TLR4 was observed for dasatinib treatment compared with LPS treatment, but total TLR4 levels were not altered (Fig. 3d, f). Taken together, these data suggest that dasatinib affects TLR4 signaling to modulate LPS-stimulated proinflammatory cytokine levels.

### Dasatinib significantly reduces LPS-induced AKT/ERK signaling in BV2 microglial cells

Several studies have shown that AKT and ERK signaling play important roles in LPS-induced inflammation [18, 19]. To examine whether dasatinib itself can regulate AKT and ERK signaling, BV2 microglial cells were treated with dasatinib (250 nM) or vehicle (1% DMSO) for 45 min followed by PBS for 45 min, and western blotting was conducted with anti-p-ERK/ERK and anti-p-AKT/AKT antibodies. We found that dasatinib itself significantly reduced p-ERK (Additional file 1: Figure S5a–c) and p-AKT (Additional file 1: Figure S5d–f) levels in BV2 microglial cells, suggesting that dasatinib itself can modulate AKT and ERK signaling.

**Fig. 4:**
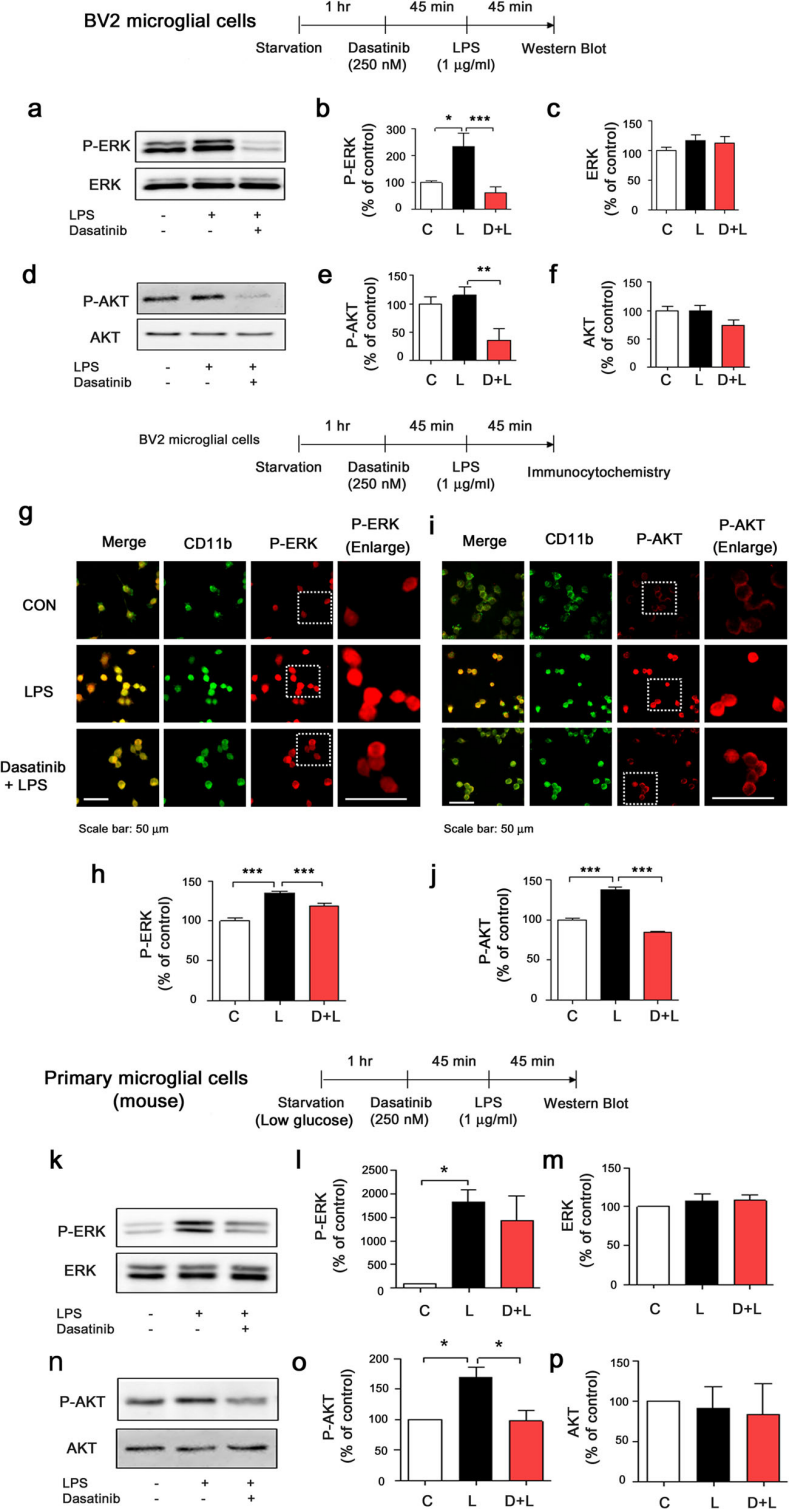
Dasatinib significantly suppresses LPS-induced AKT and/or ERK phosphorylation. **a** BV2 microglial cells were treated with vehicle (1% DMSO) or dasatinib (250 nM) for 45 min followed by PBS or LPS (1 ug/ml) for 45 min, and western blotting was performed with anti-p-ERK and anti-ERK antibodies. **b**, **c** Quantification of the data in **a** (p-ERK and ERK: con, *n* = 18; LPS, *n* = 18; dasatinib+LPS, *n* = 18). **d** BV2 microglial cells were treated with vehicle (1% DMSO) or dasatinib (250 nM) for 45 min followed by PBS or LPS (1 ug/ml) for 45 min, and western blotting was conducted with anti-p-AKT and anti-AKT antibodies. **e**, **f** Quantification of the data in **d** (p-AKT: con, *n* = 8; LPS, *n* = 8; dasatinib+LPS, *n* = 8 and AKT: con, *n* = 17; LPS, *n* = 17; dasatinib+LPS, *n* = 17). **g** BV2 microglial cells were treated with dasatinib (250 nM) or vehicle (1% DMSO) for 45 min followed by LPS (1 ug/ml) or PBS for 45 min, and immunocytochemistry was conducted with anti-p-ERK and anti-CD11b antibodies. **h** Quantification of the data in g (p-ERK: con, *n* = 239; LPS, *n* = 243; dasatinib+LPS, *n* = 212). **i** BV2 microglial cells were treated with dasatinib (250 nM) or vehicle (1% DMSO) for 45 min followed by LPS (1 ug/ml) or PBS for 45 min, and immunocytochemistry was conducted with anti-p-AKT and anti-CD11b antibodies. **j** Quantification of the data in **i** (p-AKT: con, *n* = 599; LPS, *n* = 654; dasatinib+LPS, *n* = 601). **k** Primary microglial cells were treated with vehicle (1% DMSO) or dasatinib (250 nM) for 45 min followed by PBS or LPS (1 ug/ml) for 45 min, and western blotting was performed with anti-p-ERK and anti-ERK antibodies. **l**, **m** Quantification of the data in **k** (p-ERK and ERK: con, *n* = 3; LPS, *n* = 3; dasatinib+LPS, *n* = 3). **n** Primary microglial cells were treated with vehicle (1% DMSO) or dasatinib (250 nM) for 45 min followed by PBS or LPS (1 ug/ml) for 45 min, and western blotting was conducted with anti-p-AKT and anti-AKT antibodies. **o**, **p** Quantification of the data in **n** (p-AKT and AKT: con, *n* =3; LPS, *n* = 3; dasatinib+LPS, *n* = 3). One-way ANOVA with Tukey’s post hoc test was used to analyze significant differences. *p < 0.05, **p < 0.01, ***p < 0.001

We then investigated whether dasatinib can modulate LPS-induced AKT and ERK signaling. BV2 microglial cells were treated with dasatinib (250 nM) or vehicle (1% DMSO) for 45 min followed by LPS (1 μg/ml) or PBS for 45 min, and western blotting was conducted with anti-p-ERK/ERK, anti-p-AKT/AKT, or anti-p-P38/P38 antibodies. Dasatinib significantly downregulated LPS-stimulated p-ERK levels compared to LPS treatment, but total ERK levels were not altered (Fig. 4a–c). In addition, dasatinib significantly reduced LPS-stimulated p-AKT levels compared with LPS treatment (Fig. 4d, e) without changing total AKT levels (Fig. 4d, f). However, dasatinib did not significantly alter LPS-induced p-P38 levels in BV2 microglial cells (Additional file 1: Figure S5g–i).

To further confirm our findings, BV2 microglial cells were treated with dasatinib (250 nM) or vehicle (1% DMSO) for 45 min followed by LPS (1 μg/ml) or PBS for 45 min, and immunocytochemistry was conducted with anti-p-ERK and anti-CD11b or anti-p-AKT and anti-CD11b antibodies. Dasatinib significantly downregulated LPS-induced p-ERK (Fig. 4g–h) and p-AKT (Fig. 4i–j) levels in BV2 microglial cells.

We then examined whether dasatinib differentially affects low-dose LPS (200 ng/ml)-induced AKT and ERK signaling. For these experiments, BV2 microglial cells were treated with vehicle (1% DMSO) or dasatinib (250 nM) for 45 min followed by LPS (200 ng/ml) or PBS for 45 min, and western blotting was performed with anti-p-ERK/ERK and anti-p-AKT/AKT antibodies. Again, dasatinib markedly decreased 200 ng/ml LPS-induced p-ERK (Additional file 1: Figure S6a, b) and p-AKT (Additional file 1: Figure S6d, e) levels in BV2 microglial cells. However, dasatinib did not alter LPS-induced ERK and AKT levels in BV2 microglial cells (Additional file 1: Figure S6c, f).

Lastly, we tested whether dasatinib alters LPS-stimulated AKT and ERK signaling in mouse primary microglial cells. Mouse primary microglial cells were treated with vehicle (1% DMSO) or dasatinib (250 nM) for 45 min followed by LPS (1 μg/ml) or PBS for 45 min, and western blotting was performed with anti-p-ERK/ERK and anti-p-AKT/AKT antibodies. We found that dasatinib treatment significantly decreased LPS-induced AKT phosphorylation in mouse primary microglial cells, but not p-ERK and total levels of ERK/AKT (Fig. 4k–q).

### Dasatinib inhibits AKT or ERK signaling to alter LPS-induced IL-6 or COX-2 mRNA levels in BV2 microglial cells

**Fig. 5:**
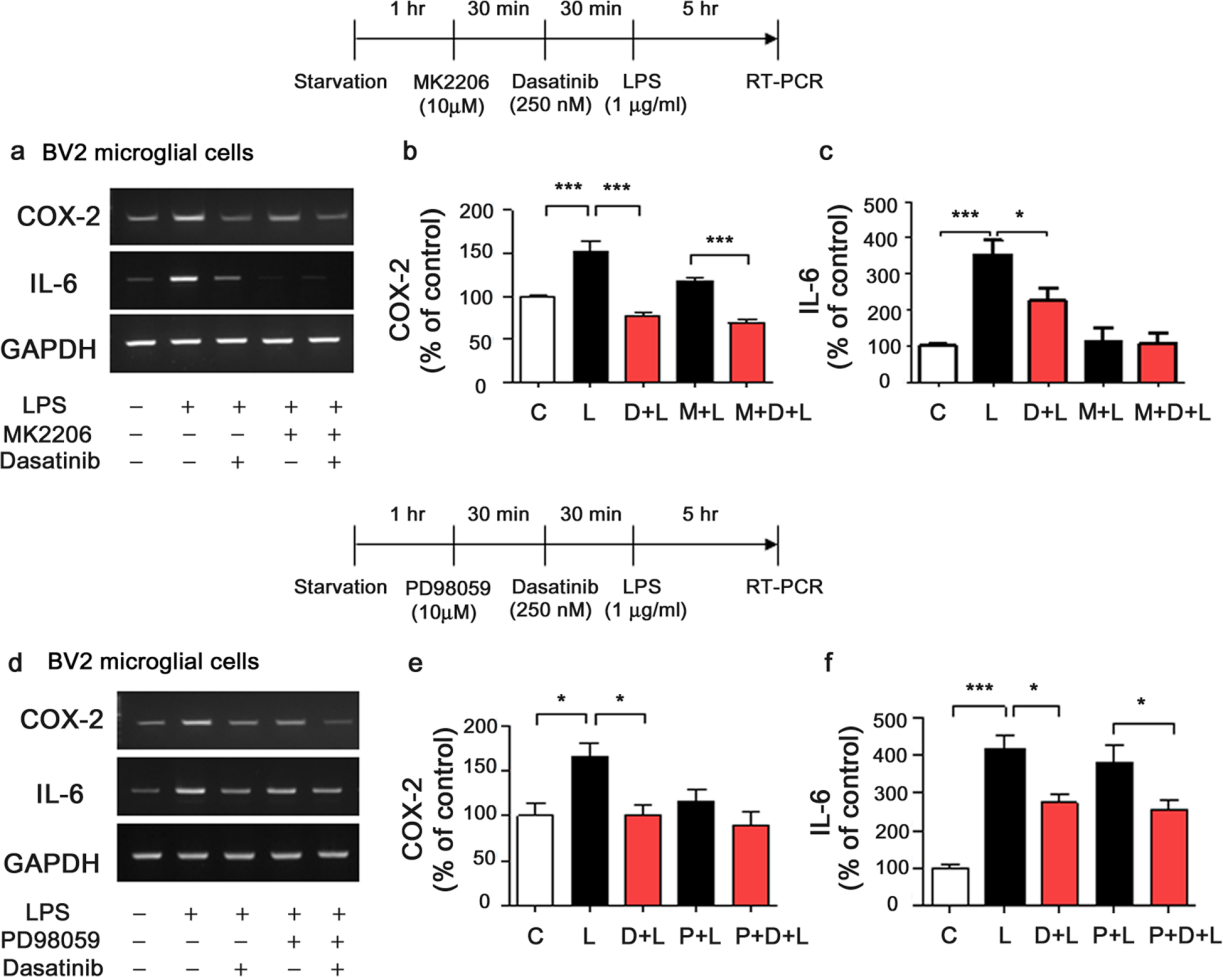
Dasatinib inhibits AKT and/or ERK phosphorylation to regulate LPS-induced proinflammatory cytokine levels in BV2 microglial cells. **a** BV2 microglial cells were treated with MK2206 (AKT inhibitor, 10 μM) or vehicle (1% DMSO) for 30 min, treated with vehicle (1% DMSO) or dasatinib (250 nM) for 30 min, and treated with PBS or LPS (1 μg/ml) for 5 h. RT-PCR was then performed to measure proinflammatory cytokine levels. **b**, **c** Quantification of the data in **a** (COX-2: con, *n* = 11; LPS, *n* = 11; dasatinib+LPS, *n* = 11; MK2206+LPS, *n* = 11; MK2206+dasatinib+LPS, *n* = 11; and IL-6: con, *n* = 8; LPS, *n* = 8; dasatinib+LPS, *n* = 8; MK2206+LPS, *n* = 8; MK2206+dasatinib+LPS, *n* = 8). **d** BV2 microglial cells were treated with PD98059 (ERK inhibitor, 10 μM) or vehicle (1% DMSO) for 30 min, treated with vehicle (1% DMSO) or dasatinib (250 nM) for 30 min, and treated with PBS or LPS (1 μg/ml) for 5 h. RT-PCR was then performed. **e**, **f** Quantification of the data in **d** (COX-2: con, *n* = 7; LPS, *n* = 7; dasatinib+LPS, *n* = 7; PD98059+LPS, *n* = 7; PD98059+dasatinib+LPS, *n* = 7; and IL-6: con, *n* = 11; LPS, *n* = 11; dasatinib+LPS, *n* = 11; PD98059+LPS, *n* = 11; PD98059+dasatinib+LPS, *n* = 11). One-way ANOVA with Tukey’s post hoc test was used to analyze significant differences. *p < 0.05, ***p < 0.001

A recent study demonstrated that the proinflammatory cytokine IL-6 is regulated by TLR4/PI3K/AKT signaling, whereas the proinflammatory cytokine COX-2 is regulated by TLR4/TRAF6/ERK signaling in myeloid cells (including monocytes, microglial cells, and macrophages) [20]. In this study, we investigated whether dasatinib downregulates AKT signaling to modulate LPS-induced COX-2 or IL-6 mRNA levels. BV2 microglial cells were pre-treated with MK2206 (AKT inhibitor, 10 μM) or vehicle (1% DMSO) for 30 min, treated with dasatinib (250 nM) or vehicle (1% DMSO) for 30 min, and treated with LPS (1 μg/ml) or PBS for 5 h. COX-2 and IL-6 mRNA levels were then measured by RT-PCR. Treatment with MK2206, dasatinib, and LPS (labeled as M+D+L) further decreased LPS-induced COX-2 mRNA levels compared with treatment with MK2206 and LPS (labeled as M+L), suggesting that dasatinib downregulates LPS-induced COX-2 mRNA levels in an AKT-independent manner (Fig. 5a, b). By contrast, MK2206, dasatinib, and LPS (M+D+L) treatment did not significantly reduce LPS-stimulated IL-6 mRNA levels compared with treatment with dasatinib and LPS (D+L) or MK2206 and LPS (M+L) (Fig. 5a, c). These data suggest that dasatinib downregulates LPS-induced IL-6 mRNA levels by inhibiting AKT signaling.

In addition, we investigated whether dasatinib regulates ERK signaling to suppress LPS-stimulated COX-2 or IL-6 mRNA levels. BV2 microglial cells were pre-treated with PD98059 (ERK inhibitor, 10 μM) or vehicle (1% DMSO) for 30 min, treated with dasatinib (250 nM) or vehicle (1% DMSO) for 30 min, and treated with LPS (1 μg/ml) or PBS for 5 h. Treatment with PD98059, dasatinib, and LPS (labeled as P+D+L) did not alter LPS-induced COX-2 mRNA levels compared with treatment with PD98059 and LPS (labeled as P+L) (Fig. 5d, e). This result indicates that dasatinib modulates ERK signaling to inhibit LPS-induced COX-2 mRNA levels. However, PD98059, dasatinib, and LPS (P+D+L) treatment further decreased IL-6 mRNA levels compared with treatment with PD98059 and LPS (P+L), suggesting that dasatinib decreases LPS-induced IL-6 mRNA levels in an ERK-independent manner (Fig. 5d, f). Taken together, these results suggest that dasatinib inhibits TLR4/ERK signaling to decrease LPS-induced COX-2 mRNA levels and suppresses TLR4/AKT signaling to downregulate LPS-induced IL-6 mRNA levels, suggesting that dasatinib affects the regulation of the mRNA levels of the LPS-induced proinflammatory cytokines COX-2 and IL-6 mRNA via multi-directional signaling pathways.

### Dasatinib reduces LPS-stimulated cytosolic and nuclear p-STAT3 levels in BV2 microglial cells

**Fig. 6:**
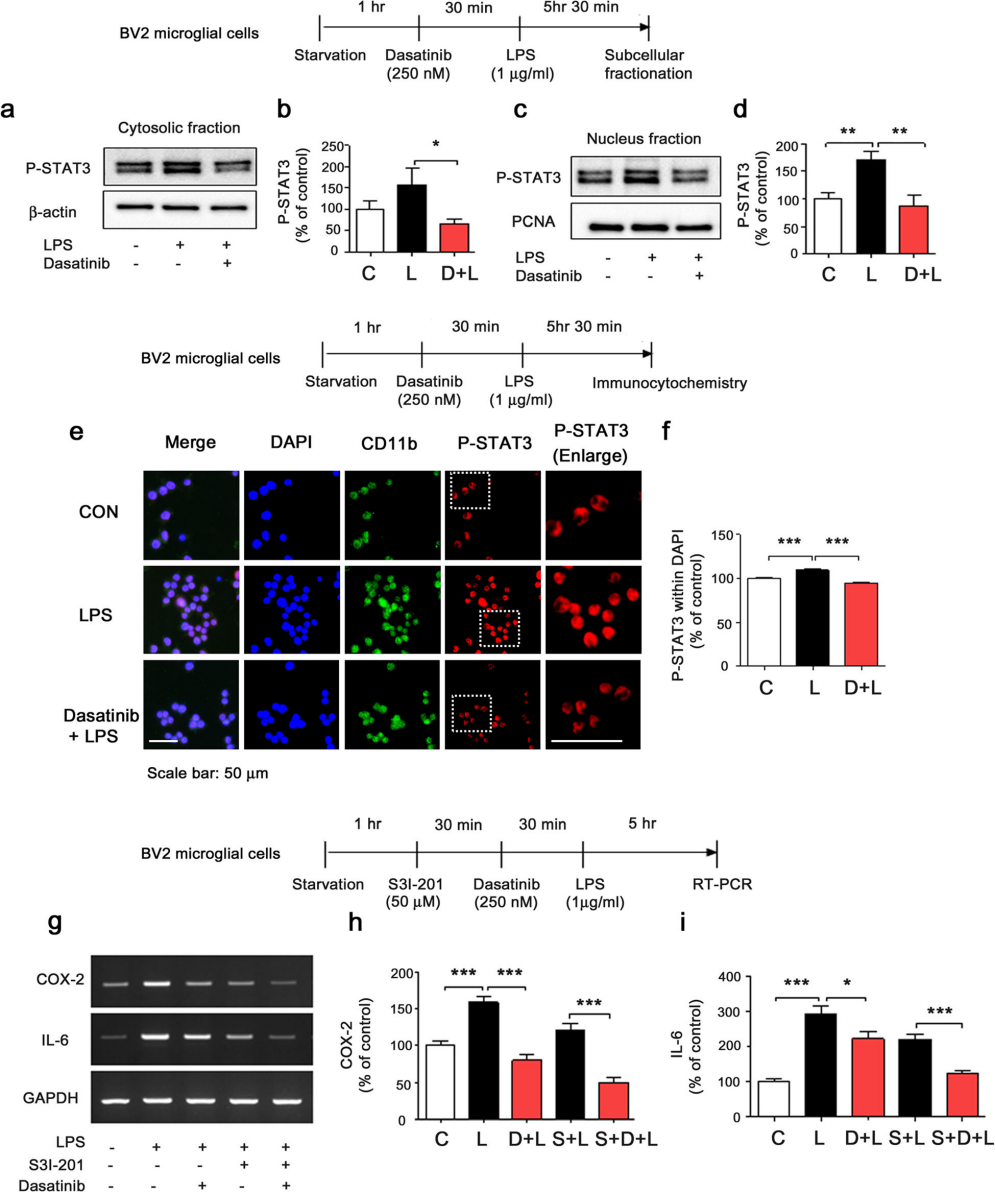
Dasatinib reduces LPS-induced cytosolic and nuclear p-STAT3 levels in BV2 microglial cells. **a** BV2 microglial cells were treated with vehicle (1% DMSO) or dasatinib (250 nM) for 30 min followed by PBS or LPS (1 μg/ml) for 5 h 30 min, and western blotting of the cytosolic fraction was performed with anti-p-STAT3 (Ser727) and anti-β-actin (as a cytosolic marker) antibodies. **b** Quantification of the data in **a** (con, *n* = 12, LPS, *n* = 12, dasatinib+LPS, *n* = 12). **c** BV2 microglial cells were treated with vehicle (1% DMSO) or dasatinib (250 nM) for 30 min followed by PBS or LPS (1 μg/ml) for 5.5 h, and western blotting of the nuclear fraction was conducted with anti-p-STAT3 (Ser727) and anti-PCNA (as a nuclear marker) antibodies. **d** Quantification of the data in **c** (con, *n* = 21; LPS, *n* = 21; dasatinib+LPS, *n* = 21). **e** BV2 microglial cells were treated with vehicle (1% DMSO) or dasatinib (250 nM) for 30 min followed by PBS or LPS (1 μg/ml) for 5.5 h, and immunostaining was performed with anti-p-STAT3 (Ser 727) and anti-CD11b antibodies. **f** Quantification of the data in **e** (con, *n* = 437; LPS, *n* = 527; dasatinib+LPS, *n* = 338). **g** BV2 microglial cells were treated with S3I-201 (STAT3 inhibitor, 50 μM) or vehicle (1% DMSO) for 30 min, treated with vehicle (1% DMSO) or dasatinib (250 nM) for 30 min, and treated with PBS or LPS (1 μg/ml) for 5 h. RT-PCR was then performed to measure proinflammatory cytokine levels. **h**, **i** Quantification of the data in **g** (COX-2: con, *n* = 26; LPS, *n* = 26; dasatinib+LPS, *n* = 26; S3I-201+LPS, *n* = 26; S3I-201+dasatinib+LPS, *n* = 26; and IL-6: con, *n* = 30; LPS, *n* = 30; dasatinib+LPS, *n* = 30; S3I-201+LPS, *n* = 30; S3I-201+dasatinib+LPS, *n* = 30). One-way ANOVA with Tukey’s post hoc test was used to analyze significant differences. *p < 0.05, **p < 0.01, ***p < 0.001

We and others have found that the transcription factor STAT3 modulates LPS-induced proinflammatory cytokine levels [17, 21, 22]. Thus, we tested whether dasatinib alters cytosolic and nuclear LPS-stimulated STAT3 signaling. For these experiments, BV2 microglial cells were treated with dasatinib (250 nM) or vehicle (1% DMSO) for 30 min followed by LPS (1 μg/ml) or PBS for 5 h 30 min, and subcellular fractionation was performed. Dasatinib significantly decreased LPS-induced cytosolic and nuclear p-STAT3 (Ser727) levels compared with LPS treatment (Fig. 6a–d).

To further confirm our findings, BV2 microglial cells were treated with dasatinib (250 nM) or vehicle (1% DMSO) for 30 min followed by LPS (1 μg/ml) or PBS for 5.5 h, and immunocytochemistry was conducted with anti-p-STAT3 (Ser727) and anti-CD11b antibodies. Dasatinib significantly reduced LPS-induced nuclear p-STAT3 (Ser727) levels in BV2 microglial cells (Fig. 6e–f).

We then examined whether dasatinib downregulates STAT3 signaling to reduce LPS-induced proinflammatory cytokine levels. BV2 microglial cells were pre-treated with S3I-201 (STAT3 inhibitor, 50 μM) or vehicle (1% DMSO) for 30 min, treated with dasatinib (250 nM) or vehicle (1% DMSO) for 30 min, and treated with LPS (1 μg/ml) or PBS for 5 h. The mRNA levels of COX-2 and IL-6 were measured by RT-PCR. Treatment with S3I-201, dasatinib, and LPS (labeled as S+D+L) further decreased LPS-stimulated COX-2 and IL-6 mRNA levels compared with treatment with S3I-201 and LPS (labeled as S+L) (Fig. 6g–i). In addition, S+D+L treatment did not alter LPS-stimulated COX-2 or IL-6 mRNA levels compared with treatment with dasatinib and LPS (labeled as D+L) (Fig. 6g–i). These data suggest that dasatinib inhibits LPS-induced COX-2 or IL-6 mRNA levels by partially regulating STAT3 signaling and/or other transcription factor/signaling pathways (Fig. 6g–i).

### Dasatinib significantly suppresses LPS-stimulated proinflammatory cytokine COX-2 and iNOS mRNA levels in primary astrocytes

**Fig. 7:**
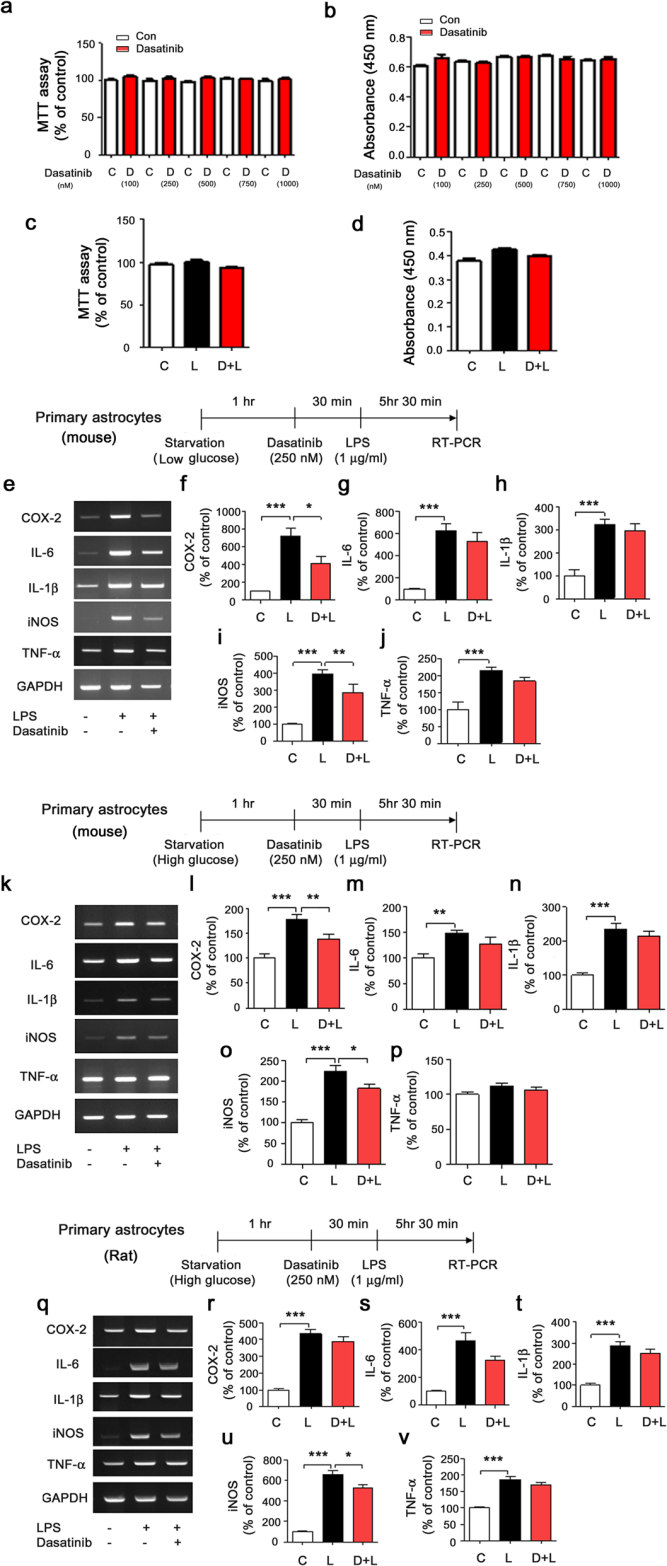
Dasatinib significantly suppresses LPS-induced proinflammatory cytokine COX-2 and iNOS mRNA levels in primary astrocytes. **a** Primary astrocyte cells were treated with vehicle (1% DMSO) or dasatinib (100, 250, 500, 750, or 1000 nM) for 6 h, and MTT assays were conducted under low-glucose conditions (100 nM, *n* = 6; 250 nM, *n* = 6; 500 nM, *n* = 6; 750 nM, *n* = 6; or 1000 nM, *n* = 6). **b** Primary astrocyte cells were treated with vehicle (1% DMSO) or dasatinib (100, 250, 500, 750, or 1000 nM) for 6 h, and BrdU proliferation assays were conducted under low-glucose conditions (100 nM, *n* = 6; 250 nM, *n* = 6; 500 nM, *n* = 6; 750 nM, *n* = 6; or 1000 nM, *n* = 6). **c** Primary astrocytes were treated with dasatinib (250 nM) or vehicle (1% DMSO) for 30 min followed by LPS (200 ng/ml) or PBS for 6 h, and MTT assays were performed under low-glucose conditions (con, *n* = 6; LPS, *n* = 6; dasatinib+LPS, *n* = 6). **d** Primary astrocytes were treated with dasatinib (250 nM) or vehicle (1% DMSO) for 30 min followed by LPS (200 ng/ml) or PBS for 6 h, and BrdU proliferation assays were performed under low-glucose conditions (con, *n* = 6; LPS, *n* = 6; dasatinib+LPS, *n* = 6). **e** Mouse primary astrocytes were treated with dasatinib (250 nM) or vehicle (1% DMSO) for 30 min followed by LPS (1 μg/ml) or PBS for 5.5 h, and proinflammatory cytokine levels were measured by RT-PCR under low-glucose conditions. **f**–**j** Quantification of the data in **e** (COX2, IL-6, IL-1β, iNOS, and TNF-alpha: con, *n* = 8; LPS, *n* = 8; dasatinib+LPS, *n* = 8). **k** Mouse primary astrocytes were treated with dasatinib (250 nM) or vehicle (1% DMSO) for 30 min followed by LPS (1 μg/ml) or PBS for 5.5 h, and proinflammatory cytokine levels were measured by RT-PCR under high-glucose conditions. **l**–**p** Quantification of the data in **k** (COX2, IL-6, IL-1β, iNOS, and TNF-alpha: con, *n* = 10; LPS, *n* = 10; dasatinib+LPS, *n* = 10). **q** Rat primary astrocytes were treated with dasatinib (250 nM) or vehicle (1% DMSO) for 30 min followed by LPS (1 μg/ml) or PBS for 5.5 h, and proinflammatory cytokine levels were measured by RT-PCR under high-glucose conditions. **r**–**v** Quantification of the data in **q** (COX2, IL-6, IL-1β, iNOS, and TNF-alpha: con, *n* = 20; LPS, *n* = 20; dasatinib+LPS, *n* = 20). Two-tailed *t* tests (**a**, **b**) and one-way ANOVA with Tukey’s post hoc test (**c**–**v**) were used to analyze significant differences. *p < 0.05, **p < 0.01, ***p < 0.001

To examine whether dasatinib can alter LPS-induced proinflammatory cytokine levels in primary astrocytes, we first examined whether dasatinib itself affects viability and proliferation in primary astrocytes using MTT and BrdU assays. For these experiments, primary astrocytes were treated with vehicle (1% DMSO) or dasatinib (100, 250, 500, 750, or 1000 nM) for 6 h, and MTT assays or BrdU proliferation assays were performed. Dasatinib did not alter cell viability and cell proliferation up to 1000 nM in mouse primary astrocytes (Fig. 7a, b). We then investigated whether dasatinib-treated mouse primary astrocytes affect cell viability and proliferation in the presence of LPS. Mouse primary astrocytes were treated with vehicle (1% DMSO) or dasatinib (250 nM) for 30 min followed by LPS (200 ng/ml) or PBS for 5.5 h, and MTT and BrdU assays were conducted. We found that dasatinib did not exhibit astrocytic cytotoxicity and did not alter cell proliferation in the presence of LPS (Fig. 7c, d).

To determine whether dasatinib alters LPS-stimulated proinflammatory cytokine levels under low-glucose conditions, mouse primary astrocytes were treated with vehicle (1% DMSO) or dasatinib (250 nM) for 30 min followed by LPS (1 μg/ml) or PBS for 5.5 h. Proinflammatory cytokine levels were then measured by RT-PCR under low-glucose conditions. Interestingly, dasatinib significantly suppressed LPS-induced COX-2 and iNOS mRNA levels in mouse primary astrocytes under low-glucose conditions (Fig. 7e–j).

To test whether dasatinib can differentially regulate LPS-induced neuroinflammation under high-glucose conditions, mouse primary astrocytes were treated with vehicle (1% DMSO) or dasatinib (250 nM) for 30 min followed by LPS (1 μg/ml) or PBS for 5.5 h, and proinflammatory cytokine levels were measured by RT-PCR. We found that dasatinib treatment significantly reduced LPS-induced COX-2 and iNOS mRNA levels in mouse primary astrocytes under high-glucose conditions (Fig. 7k–p). In addition, dasatinib treatment significantly reduced only LPS-stimulated iNOS mRNA levels and not the levels of other proinflammatory cytokines in rat primary astrocytes under high-glucose conditions (Fig. 7q–v). These data suggest that dasatinib differentially affects LPS-induced neuroinflammatory responses in primary astrocytes depending on the species (rat vs mouse). Based on our data, we used low-glucose conditions for subsequent experiments to determine the effects of dasatinib on LPS-induced neuroinflammatory responses in primary astrocytes.

We then examined whether dasatinib modulates anti-inflammatory responses. Mouse primary astrocytes were treated with vehicle (1% DMSO) or dasatinib (250 nM) for 30 min followed by LPS (1 μg/ml) or PBS for 5.5 h, and anti-inflammatory mediator levels were measured by real-time PCR under low-glucose conditions. We found that LPS treatment significantly increased anti-inflammatory cytokine IL-10 mRNA expression, whereas IL-4 mRNA levels exhibited a tendency to increase in mouse primary astrocytes (Additional file 1: Figure S7a–c). Dasatinib significantly increased anti-inflammatory cytokine IL-10 mRNA levels, and a trend toward increased IL-4 mRNA levels was observed in LPS-treated mouse primary astrocytes pre-treated with dasatinib (Additional file 1: Figure S7a–c). These data suggest that dasatinib modulates LPS-stimulated neuroinflammation by upregulating anti-inflammatory responses in primary astrocytes.

### Dasatinib decreases LPS-stimulated AKT signaling and nuclear STAT3 phosphorylation in primary astrocytes

Since we observed that dasatinib affects AKT/ERK signaling in BV2 microglial cells (Fig. 4), we tested whether dasatinib modulates AKT and ERK phosphorylation in primary astrocytes. For these experiments, mouse primary astrocytes were treated with dasatinib (250 nM) or vehicle (1% DMSO) for 45 min followed by treatment with PBS for 45 min. Then, western blotting was conducted with anti-p-ERK/ERK and anti-p-AKT/AKT antibodies. Dasatinib itself markedly suppressed p-ERK and p-AKT/AKT levels in mouse primary astrocytes compared to vehicle treatment (Additional file 1: Figure S8a–f).

**Fig. 8:**
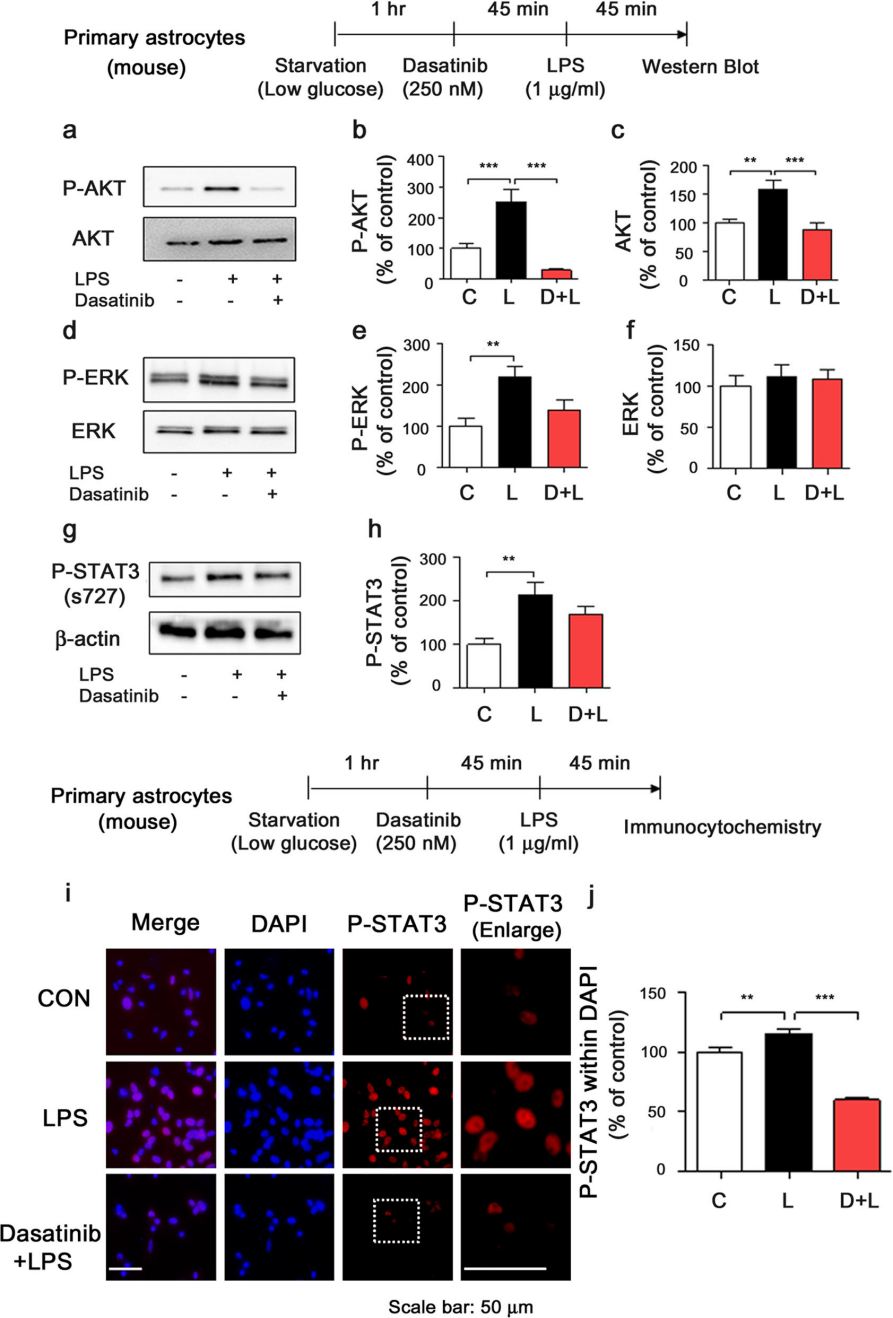
Dasatinib significantly decreases LPS-induced AKT phosphorylation in primary astrocytes. **a**, **d**, **g** Mouse primary astrocytes were treated with dasatinib (250 nM) or vehicle (1% DMSO) for 45 min followed by LPS (1 μg/ml) or PBS for 45 min, and western blotting was conducted with anti-p-AKT/AKT, anti-p-ERK/ERK, or anti-p-STAT3/STAT3 antibodies under low-glucose conditions. **b**, **c**; **e**, **f**; **h** Quantification of the data in **a** (con, *n* = 10; LPS, *n* = 10; dasatinib+LPS, *n* = 10), **d** (con, *n* = 10; LPS, *n* = 10, dasatinib+LPS, *n* = 10), or **g** (con, *n* = 6; LPS, *n* = 6; dasatinib+LPS, *n* = 6). **i** Mouse primary astrocytes were treated with dasatinib (250 nM) or vehicle (1% DMSO) for 45 min followed by LPS (1 μg/ml) or PBS for 45 min, and immunostaining was performed with an anti-p-STAT3 antibody. **j** Quantification of the data in **i** (con, *n* = 622; LPS, *n* = 670; dasatinib+LPS, *n* = 463). One-way ANOVA with Tukey’s post hoc test was used to analyze significant differences. **p < 0.01, ***p < 0.001

To examine the effects of dasatinib on LPS-induced AKT and ERK signaling in primary astrocytes, mouse primary astrocytes were treated with vehicle (1% DMSO) or dasatinib (250 nM) for 45 min followed by LPS (1 μg/ml) or PBS for 45 min, and western blotting was conducted with anti-p-AKT/AKT, anti-p-ERK/ERK, or anti-p-STAT3/STAT3 antibodies. Dasatinib significantly reduced LPS-stimulated p-AKT and AKT levels (Fig. 8a–c), with a trend toward decreased LPS-induced p-ERK levels (Fig. 8d, e), but the total ERK level was not altered (Fig. 8d, f). In addition, dasatinib did not significantly reduce LPS-stimulated P38 phosphorylation in mouse primary astrocytes compared to LPS treatment (Additional file 1: Figure S8g–i). Moreover, a trend toward decreased LPS-induced STAT3 phosphorylation was observed in dasatinib-treated mouse primary astrocytes (Fig. 8g, h).

To further determine whether dasatinib can alter LPS-induced nuclear p-STAT3 levels in primary glial cells, mouse primary astrocytes were treated with vehicle (1% DMSO) or dasatinib (250 nM) for 45 min followed by LPS (1 μg/ml) or PBS for 45 min, and immunocytochemistry was performed with an anti-p-STAT3 (s727) antibody. We observed that LPS treatment significantly increased LPS-induced nuclear p-STAT3 levels compared with the vehicle treatment (Fig. 8i–j). Dasatinib treatment significantly decreased LPS-stimulated nuclear p-STAT3 levels in mouse primary astrocytes compared with LPS treatment (Fig. 8i, j).

### Intraperitoneal administration of dasatinib inhibits LPS-stimulated microglial and astrocyte activation in wild-type mice

**Fig. 9:**
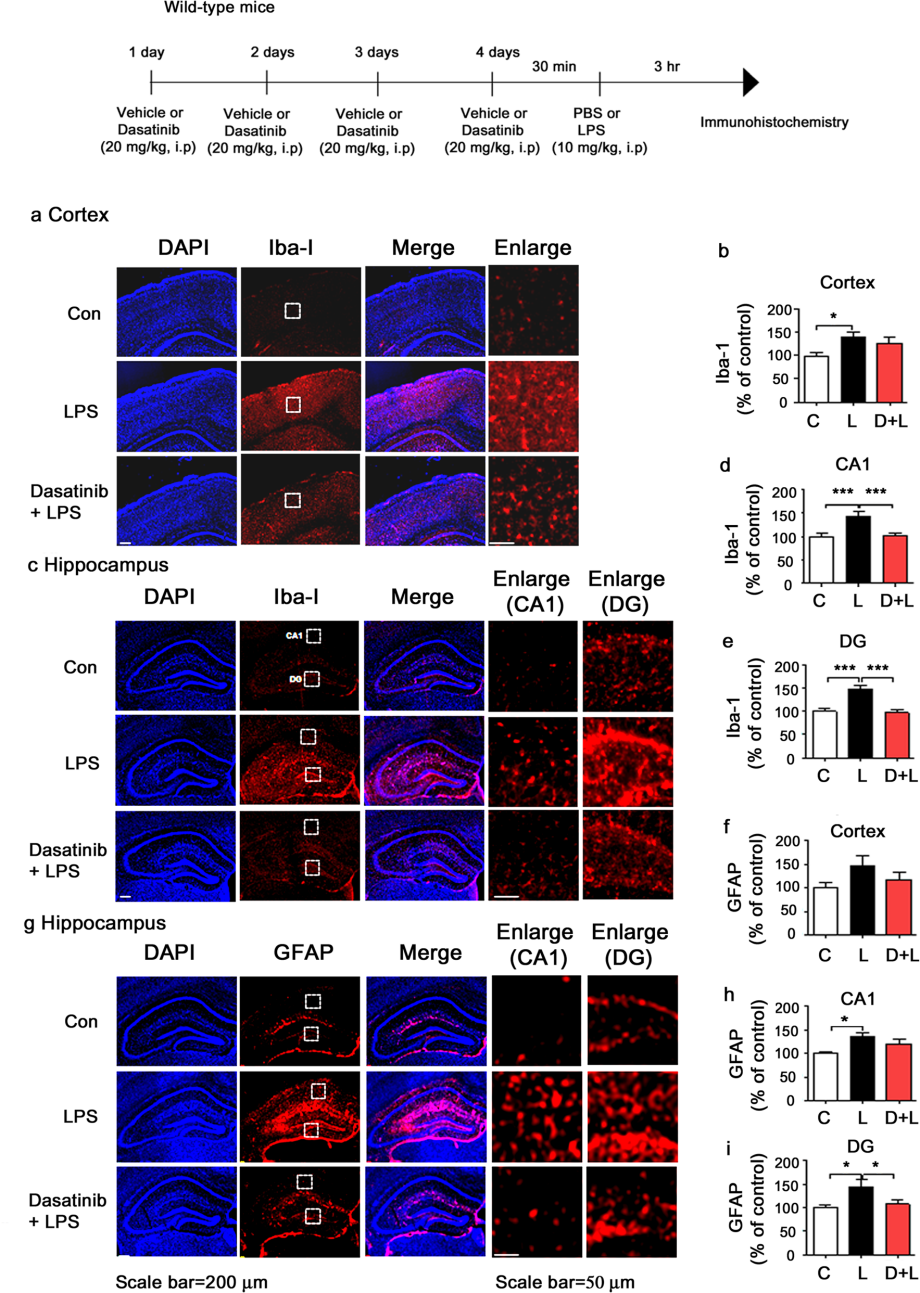
Intraperitoneal injection of dasatinib significantly reduces LPS-stimulated Iba-1 and GFAP immunoreactivity in wild-type mice. **a**, **c** Wild-type mice were injected with dasatinib (20 mg/kg, i.p.) or vehicle (4% DMSO + 30% PEG + 5% Tween 80, i.p.) daily for 4 days, followed by injection with LPS (10 mg/kg, i.p.) or PBS. Three hours after LPS or PBS injection, immunohistochemistry was conducted with an anti-Iba-1 antibody. **b**, **d**, **e** Quantification of the data in **a** (cortex: con, *n* = 8 mice; LPS, *n* = 9 mice; dasatinib+LPS, *n* = 9 mice) and **c** (hippocampus: con, *n* = 8 mice; LPS, *n* = 9 mice; dasatinib+LPS, *n* = 9 mice). **f**, **g** Wild-type mice were injected with dasatinib (20 mg/kg, i.p.) or vehicle (4% DMSO + 30% PEG + 5% Tween 80, i.p.) daily for 4 days, followed by injection with LPS (10 mg/kg, i.p.) or PBS. Three hours after LPS or PBS injection, immunohistochemistry was conducted with an anti-GFAP antibody (cortex: con, *n* = 8 mice; LPS, *n* = 9 mice; dasatinib+LPS, *n* = 9 mice). **h**, **i** Quantification of the data in **g** (hippocampus: con, *n* = 8 mice; LPS, *n* = 9 mice; dasatinib+LPS, *n* = 9 mice). One-way ANOVA with Tukey’s post hoc test was used to analyze significant differences. *p < 0.05, ***p < 0.001

To examine whether dasatinib can affect LPS-induced microglial and astrocyte activation in vivo, wild-type mice were injected with dasatinib (20 mg/kg, i.p. daily for 4 days) or vehicle (4% DMSO + 30% PEG + 5% Tween 80) and subsequently injected with LPS (10 mg/kg, i.p.) or PBS. Three hours after LPS or PBS injection, the mice were perfused and fixed, and immunohistochemistry was performed with anti-Iba-1 or anti-GFAP antibodies. We found that LPS-injected wild-type mice exhibited microglia and astrocyte hypertrophy and significantly increased microglial and astrocyte activation compared to vehicle-injected wild-type mice (Fig. 9). Intraperitoneal administration of dasatinib significantly inhibited microglial activation in the hippocampus CA1 and DG in LPS-injected wild-type mice, but not in the cortex (Fig. 9a–e). In addition, intraperitoneal treatment with dasatinib significantly suppressed astrocyte activation in the hippocampus DG in LPS-injected wild-type mice, but not in the cortex and hippocampus CA1 (Fig. 9f–i).

### Intraperitoneal injection of dasatinib significantly downregulates LPS-induced COX-2 and IL-6 levels in wild-type mice

**Fig. 10:**
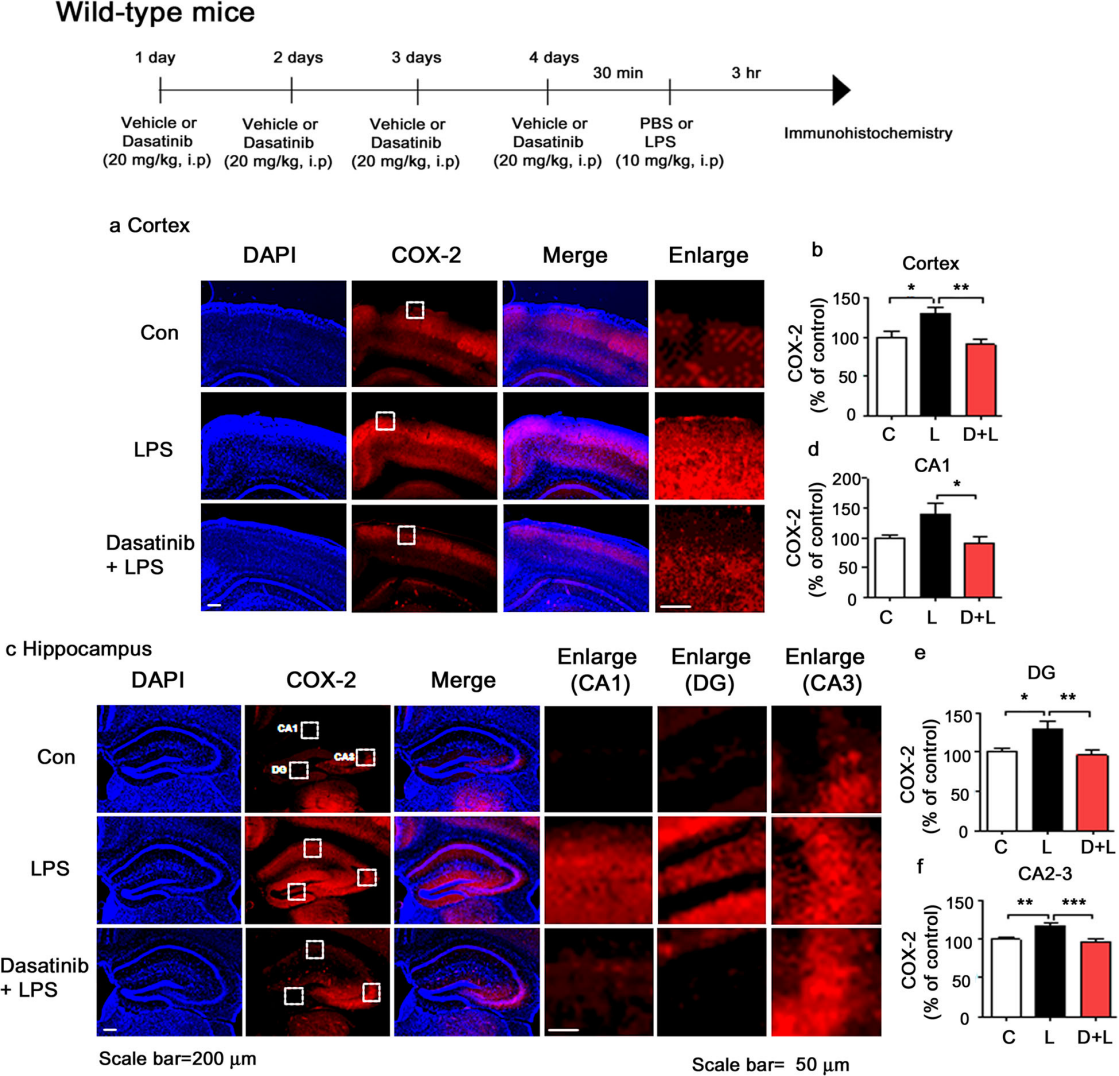
Intraperitoneal injection of dasatinib significantly downregulates LPS-stimulated COX-2 levels in wild-type mice. **a**, **c** Wild-type mice were injected with dasatinib (20 mg/kg, i.p.) or vehicle (4% DMSO + 30% PEG + 5% Tween 80, i.p.) daily for 4 days, followed by injection with LPS (10 mg/kg, i.p.) or PBS. Three hours after LPS or PBS injection, immunohistochemistry was conducted with an anti-COX-2 antibody. **b**, **d**–**f** Quantification of the data in **a** and **c** (cortex: con, *n* = 8 mice; LPS, *n* = 9 mice; dasatinib+LPS, *n* = 9 mice; and hippocampus: con, *n* = 8 mice; LPS, *n* = 9 mice; dasatinib+LPS, *n* = 9 mice). One-way ANOVA with Tukey’s post hoc test was used to analyze significant differences. *p < 0.05, **p < 0.01, ***p < 0.001

**Fig. 11:**
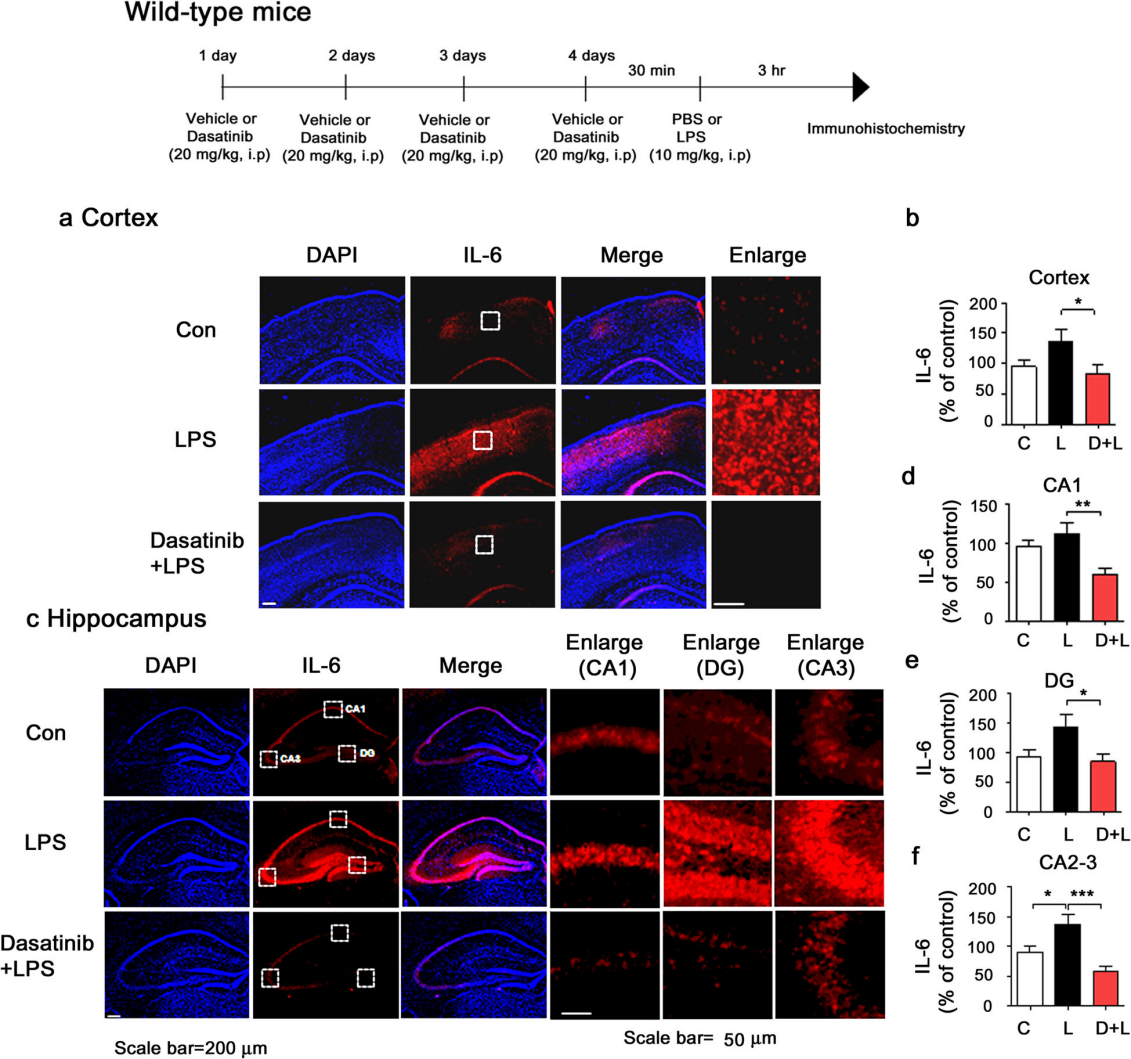
Intraperitoneal injection of dasatinib significantly downregulates LPS-stimulated IL-6 levels in wild-type mice. **a**, **c** Wild-type mice were injected with dasatinib (20 mg/kg, i.p.) or vehicle (4% DMSO + 30% PEG + 5% Tween 80, i.p.) daily for 4 days, followed by injection with LPS (10 mg/kg, i.p.) or PBS for 3 h. After 3 h, immunohistochemistry was conducted with an anti-IL-6 antibody. **b**, **d**–**f** Quantification of the data shown in **a** (cortex: con, *n* = 8 mice; LPS, *n* = 9 mice; dasatinib+LPS, *n* = 9 mice) and **c** (hippocampus: con, *n* = 8 mice; LPS, *n* = 9 mice; dasatinib+LPS, *n* = 9 mice). One-way ANOVA with Tukey’s post hoc test was used to analyze significant differences. *p < 0.05, **p < 0.01, ***p < 0.001

To examine whether dasatinib can affect LPS-induced COX-2 and IL-6 levels in vivo, wild-type mice were injected with dasatinib (20 mg/kg, i.p. daily for 4 days) or vehicle (4% DMSO + 30% PEG + 5% Tween 80) and subsequently injected with LPS (10 mg/kg, i.p.) or PBS. Three hours after LPS or PBS injection, the mice were perfused and fixed, and immunohistochemistry was performed with anti-COX-2 or anti-IL-6 antibodies. LPS-injected wild-type mice exhibited increases in COX-2 and IL-6 levels compared to vehicle-injected wild-type mice (Figs. 10 and 11). Importantly, intraperitoneal injection with dasatinib markedly suppressed COX-2 and IL-6 levels in the cortex and the hippocampus CA1, DG, and CA2-3 in LPS-injected wild-type mice (Figs. 10a–f and 11a–f). These data suggest that intraperitoneal injection of dasatinib regulates LPS-induced proinflammatory cytokine COX-2 and IL-6 levels in wild-type mice.

### Oral administration of dasatinib suppresses LPS-evoked microglial and astrocyte activation in wild-type mice

**Fig. 12:**
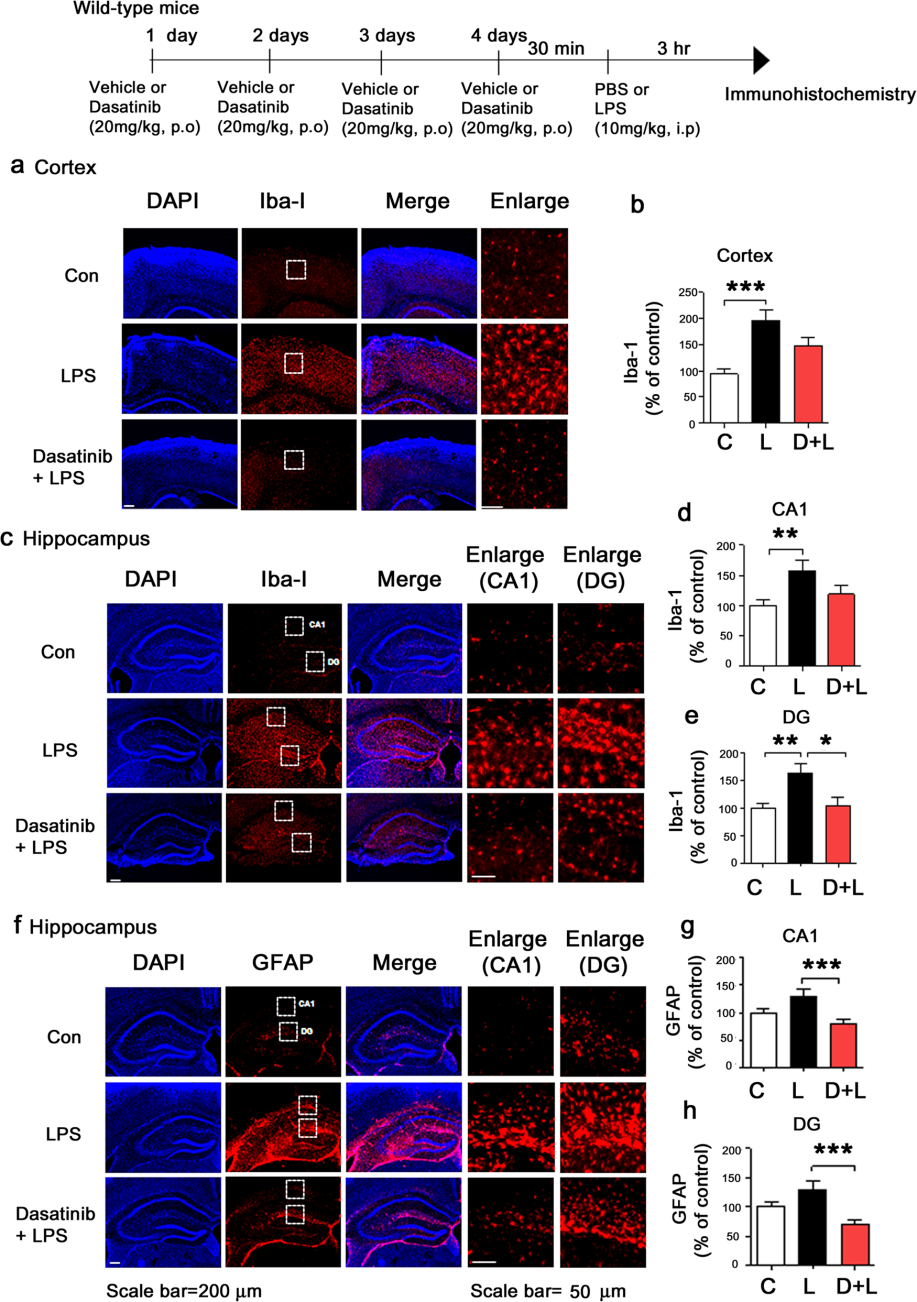
Oral administration of dasatinib daily for 4 days markedly suppresses LPS-stimulated Iba-1 and GFAP immunoreactivity in wild-type mice. **a**, **c** Wild-type mice were injected with dasatinib (20 mg/kg, p.o.) or vehicle (4% DMSO + 30% PEG + 5% Tween 80, p.o.) daily for 4 days, followed by injection with LPS (10 mg/kg, i.p.) or PBS. Three hours after LPS or PBS injection, immunohistochemistry was conducted with an anti-Iba-1 antibody. **b**, **d**, **e** Quantification of the data in **a** (cortex: con, *n* = 8 mice; LPS, *n* = 8 mice; dasatinib+LPS, *n* = 8 mice) and **c** (hippocampus: con, *n* = 8 mice; LPS, *n* = 8 mice; dasatinib+LPS, *n* = 8 mice). **f** Wild-type mice were injected with dasatinib (20 mg/kg, p.o.) or vehicle (4% DMSO + 30% PEG + 5% Tween 80, p.o.) daily for 4 days, followed by injection with LPS (10 mg/kg, i.p.) or PBS. Three hours after LPS or PBS injection, immunohistochemistry was conducted with an anti-GFAP antibody. **g**, **h** Quantification of the data in **f** (hippocampus: con, *n* = 8 mice; LPS, *n* = 8 mice; dasatinib+LPS, *n* = 8 mice). One-way ANOVA with Tukey’s post hoc test was used to analyze significant differences. *p < 0.05, **p < 0.01, ***p < 0.001

To investigate whether oral administration of dasatinib can regulate microglial and astrocyte activation, wild-type mice were injected with dasatinib (20 mg/kg, p.o. daily for 4 days) or vehicle (4% DMSO + 30% PEG + 5% Tween 80) and subsequently injected with LPS (10 mg/kg, i.p.) or PBS. Three hours after LPS or PBS injection, the mice were perfused and fixed, and immunohistochemistry was performed with anti-Iba-1 or anti-GFAP antibodies. LPS-injected wild-type mice exhibited microglia and astrocyte hypertrophy and increased microglial and astrocyte activation compared to vehicle-injected wild-type mice (Fig. 12). Oral administration of dasatinib daily for 4 days suppressed microglial activation in the hippocampus DG in LPS-injected wild-type mice, but not in the cortex and hippocampus CA1 (Fig. 12a–e). In addition, oral administration of dasatinib daily for 4 days significantly suppressed astrocyte activation in the hippocampus DG and CA1 in LPS-injected wild-type mice (Fig. 12f–h).

**Fig. 13:**
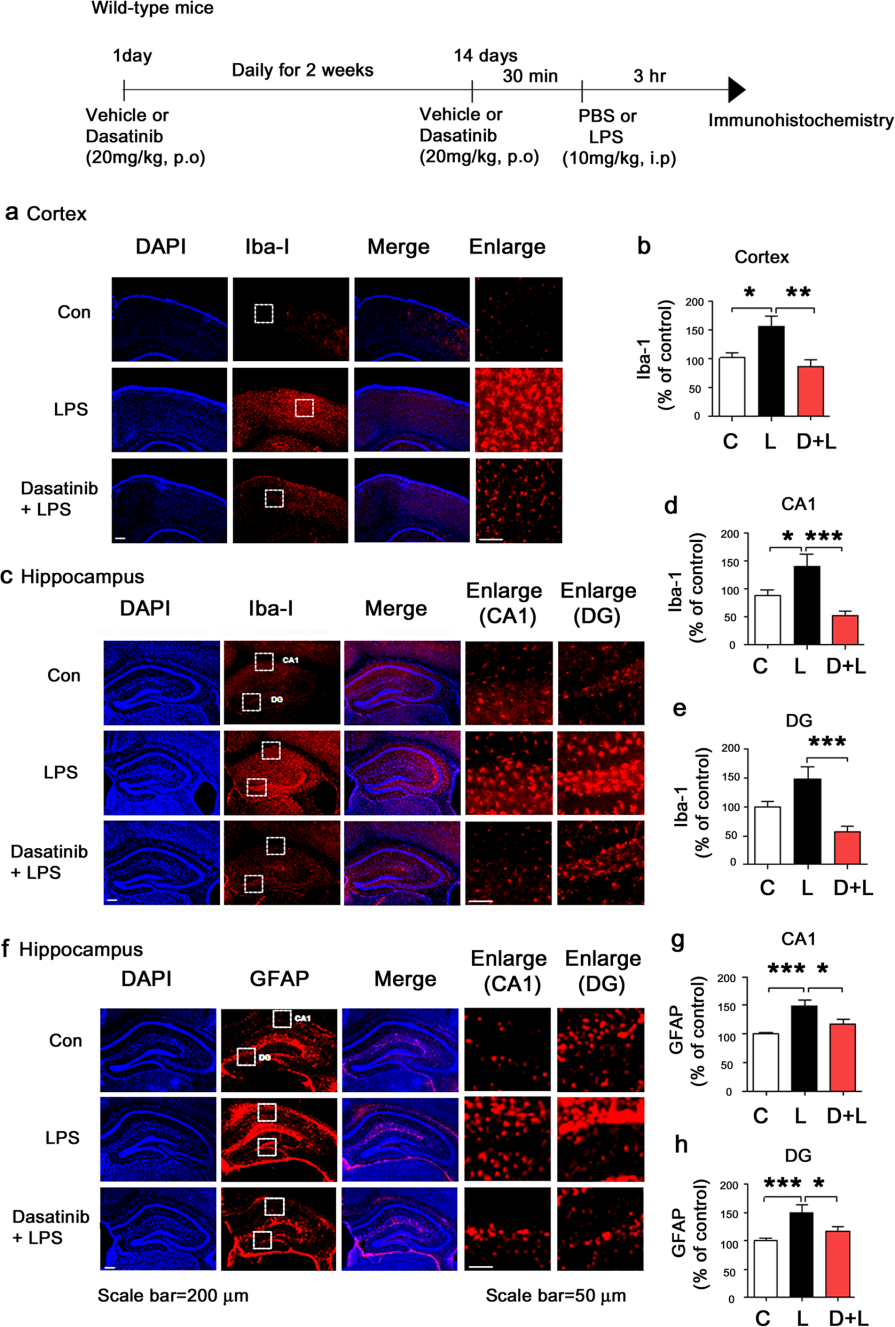
Oral administration of dasatinib daily for 2 weeks significantly decreases the LPS-induced increment of Iba-1 and GFAP immunoreactivity in wild-type mice. **a**, **c** Wild-type mice were injected with dasatinib (20 mg/kg, p.o.) or vehicle (4% DMSO + 30% PEG + 5% Tween 80, p.o.) daily for 2 weeks, followed by injection with LPS (10 mg/kg, i.p.) or PBS. Three hours after LPS or PBS injection, immunohistochemistry was conducted with an anti-Iba-1 antibody. **b** Quantification of the data in **a** (cortex: con, *n* = 8 mice; LPS, *n* = 8 mice; dasatinib+LPS, *n* = 8 mice). **d**, **e** Quantification of the data in **c** (hippocampus: con, *n* = 8 mice; LPS, *n* = 8 mice; dasatinib+LPS, *n* = 8 mice). **f** Wild-type mice were injected with dasatinib (20 mg/kg, p.o.) or vehicle (4% DMSO + 30% PEG + 5% Tween 80, p.o.) daily for 2 weeks, followed by injection with LPS (10 mg/kg, i.p.) or PBS. Three hours after LPS or PBS injection, immunohistochemistry was conducted with an anti-GFAP antibody. **g**, **h** Quantification of the data in **f** (hippocampus: con, *n* = 8 mice; LPS, *n* = 8 mice; dasatinib+LPS, *n* = 8 mice). One-way ANOVA with Tukey’s post hoc test was used to analyze significant differences. *p < 0.05, **p < 0.01, ***p < 0.001

We then examined whether longer dasatinib treatment can further regulate microglial and astrocyte activation. For these experiments, wild-type mice were orally administered dasatinib (20 mg/kg, p.o.) or vehicle (4% DMSO + 30% PEG + 5% Tween 80) daily for 2 weeks, followed by injection with LPS (10 mg/kg, i.p.) or PBS. Three hours after LPS or PBS injection, the wild-type mice were perfused and fixed, and immunohistochemistry was performed with anti-Iba-1 or anti-GFAP antibodies. Oral administration of dasatinib daily for 2 weeks significantly reduced Iba-1 and GFAP immunoreactivity in the cortex and hippocampus CA1 and DG in LPS-injected wild-type mice (Fig. 13a–e). In addition, oral administration of dasatinib daily for 2 weeks markedly inhibited LPS-induced microglial hypertrophy and Iba-1 immunoreactivity in the hippocampus CA1 and DG and cortex in wild-type mice (Fig. 13a–e). Finally, oral injection of dasatinib daily for 2 weeks significantly reduced LPS-induced astrocyte hypertrophy and GFAP immunoreactivity in the hippocampus DG and CA1 in wild-type mice (Fig. 13f–h). These data suggest that oral administration of dasatinib suppresses LPS-evoked microglia/astrocyte activation in wild-type mice.

### Administration of dasatinib by oral gavage markedly reduces LPS-induced COX-2 levels in wild-type mice

**Fig. 14:**
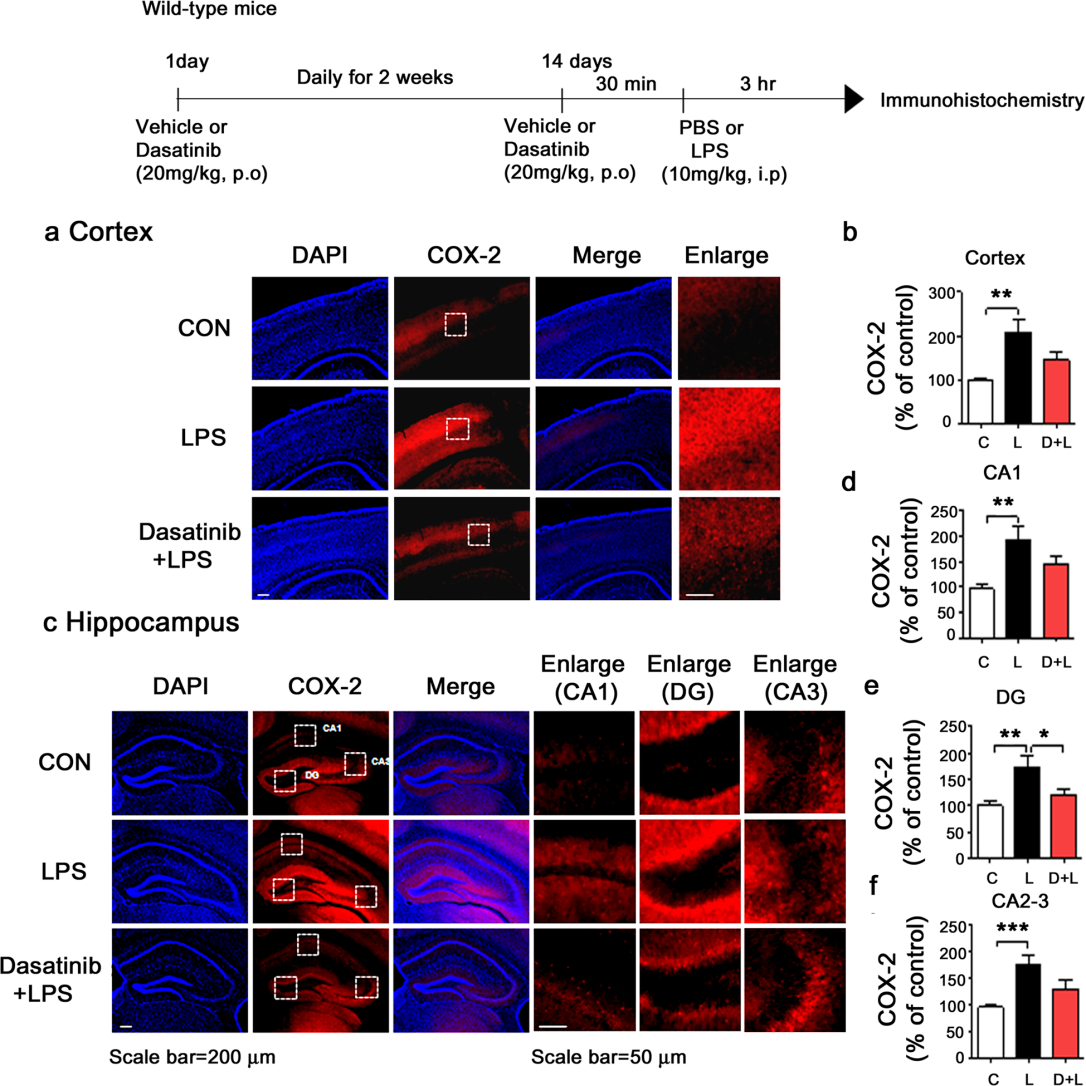
Oral administration of dasatinib daily for 2 weeks significantly downregulates LPS-stimulated IL-6 level in wild-type mice. **a**, **c** Wild-type mice were injected with dasatinib (20 mg/kg, p.o.) or vehicle (4% DMSO + 30% PEG + 5% Tween 80, i.p.) daily for 2 weeks, followed by injection with LPS (10 mg/kg, i.p.) or PBS. Three hours after LPS or PBS injection, immunohistochemistry was conducted with an anti-COX-2 antibody. **b** Quantification of the data shown in **a** (cortex: con, *n* = 8 mice; LPS, *n* = 8 mice; dasatinib+LPS, *n* = 8 mice). **d**–**f** Quantification of the data shown in **c** (hippocampus: con, *n* = 8 mice; LPS, *n* = 8 mice; dasatinib+LPS, *n* = 8 mice). One-way ANOVA with Tukey’s post hoc test was used to analyze significant differences. *p < 0.05, **p < 0.01, ***p < 0.001

To examine whether oral administration of dasatinib affects LPS-stimulated COX-2 levels, wild-type mice were treated with dasatinib (20 mg/kg, p.o. daily for 2 weeks) or vehicle (4% DMSO + 30% PEG + 5% Tween 80) and subsequently injected with LPS (10 mg/kg, i.p.) or PBS. Three hours after LPS or PBS injection, the mice were perfused and fixed, and immunohistochemistry was performed with an anti-COX-2 antibody. LPS-injected wild-type mice exhibited significant increase in COX-2 levels compared to vehicle-injected wild-type mice (Fig. 14). Oral administration of dasatinib significantly inhibited COX-2 levels in the hippocampus DG in LPS-injected wild-type mice, with a tendency toward decreased COX-2 levels in the cortex and hippocampus CA1/CA2-3 (Fig. 14a–f). These data suggest that oral injection of dasatinib downregulates LPS-induced proinflammatory cytokine COX-2 levels in wild-type mice.

### Dasatinib regulates LPS-evoked plasma proinflammatory cytokine levels in wild-type mice

**Fig. 15:**
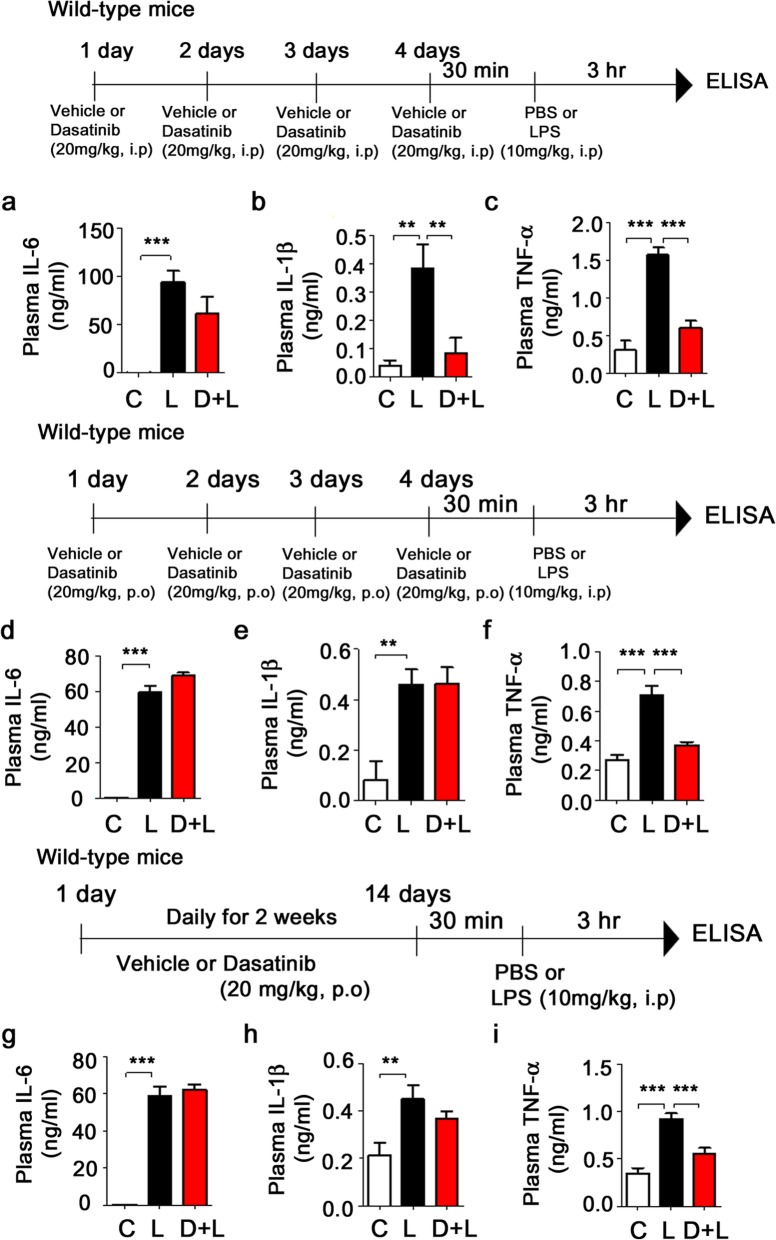
Dasatinib regulates LPS-evoked plasma proinflammatory cytokine levels in wild-type mice. **a**–**c** Wild-type mice were injected with dasatinib (20 mg/kg, i.p.) or vehicle (4% DMSO + 30% PEG + 5% Tween 80, i.p.) daily for 4 days, followed by injection with LPS (10 mg/kg, i.p.) or PBS. Three hours after LPS or PBS injection, plasma IL-6, IL-1β, and TNF-α levels were measured by ELISA (con, *n* = 8 mice; LPS, *n* = 8 mice; dasatinib+LPS, *n* = 8 mice). **d**–**f** Wild-type mice were orally administered dasatinib (20 mg/kg, p.o.) or vehicle (4% DMSO + 30% PEG + 5% Tween 80, p.o.) daily for 4 days, followed by injection with LPS (10 mg/kg, i.p.) or PBS. Three hours after LPS or PBS injection, plasma IL-6, IL-1β, and TNF-α levels were measured by ELISA (con, *n* = 8 mice; LPS, *n* = 8 mice; dasatinib+LPS, *n* = 8 mice). **g**–**i** Wild-type mice were orally administered dasatinib (20 mg/kg, p.o.) or vehicle (4% DMSO + 30% PEG + 5% Tween 80, p.o.) daily for 2 weeks, followed by injection with LPS (10 mg/kg, i.p.) or PBS. Three hours after LPS or PBS injection, plasma IL-6, IL-1β, and TNF-α levels were measured by ELISA (con, *n* = 8 mice; LPS, *n* = 8 mice; dasatinib+LPS, *n* = 8 mice). One-way ANOVA with Tukey’s post hoc test was used to analyze significant differences. **p < 0.01, ***p < 0.001

To examine whether dasatinib alters circulating proinflammatory cytokine levels, wild-type mice were injected with dasatinib (20 mg/kg, i.p. daily for 4 days) or vehicle (4% DMSO + 30% PEG + 5% Tween 80), followed by injection with LPS (10 mg/kg, i.p.) or PBS. Three hours after LPS or PBS injection, mouse blood was collected, and plasma IL-6, IL-1β, and TNF-α levels were measured by ELISA. We found that LPS-injected wild-type mice exhibited increased plasma IL-6, IL-1β, and TNF-α levels compared to vehicle-injected wild-type mice (Fig. 15a–c). Interestingly, we observed that intraperitoneal injection of dasatinib significantly decreased LPS-induced plasma TNF-α and IL-1β levels and exhibited a trend toward decreased plasma IL-6 levels (Fig. 15a–c).

In addition, we tested whether administration of dasatinib by oral gavage differentially modulates circulating proinflammatory cytokine levels. Wild-type mice were orally administered dasatinib (20 mg/kg, p.o. daily for 4 days) or vehicle (4% DMSO + 30% PEG + 5% Tween 80), followed by injection with LPS (10 mg/kg, i.p.) or PBS. Three hours after LPS or PBS injection, we collected mouse blood and measured plasma IL-6, IL-1β, and TNF-α levels using ELISA. We found that oral administration of dasatinib daily for 4 days significantly decreased plasma TNF-α levels in LPS-treated wild-type mice, but not plasma IL-1β and IL-6 levels (Fig. 15d–f).

We then examined whether longer treatment with dasatinib via oral administration can regulate circulating proinflammatory cytokine levels. For these experiments, wild-type mice were orally administered dasatinib (20 mg/kg, p.o.) or vehicle (4% DMSO + 30% PEG + 5% Tween 80) daily for 2 weeks, followed by injection with LPS (10 mg/kg, i.p.) or PBS. Again, oral administration of dasatinib daily for 2 weeks significantly decreased plasma TNF-α levels, with a trend toward decreased plasma IL-1β levels but not plasma IL-6 levels (Fig. 15g, i). These data suggest that injection of dasatinib (both i.p. and p.o.) can regulate peripheral inflammatory responses in LPS-injected wild-type mice.

### Dasatinib exhibits a tendency toward decreased LPS-induced neutrophil rolling in the CNS vasculature

**Fig. 16:**
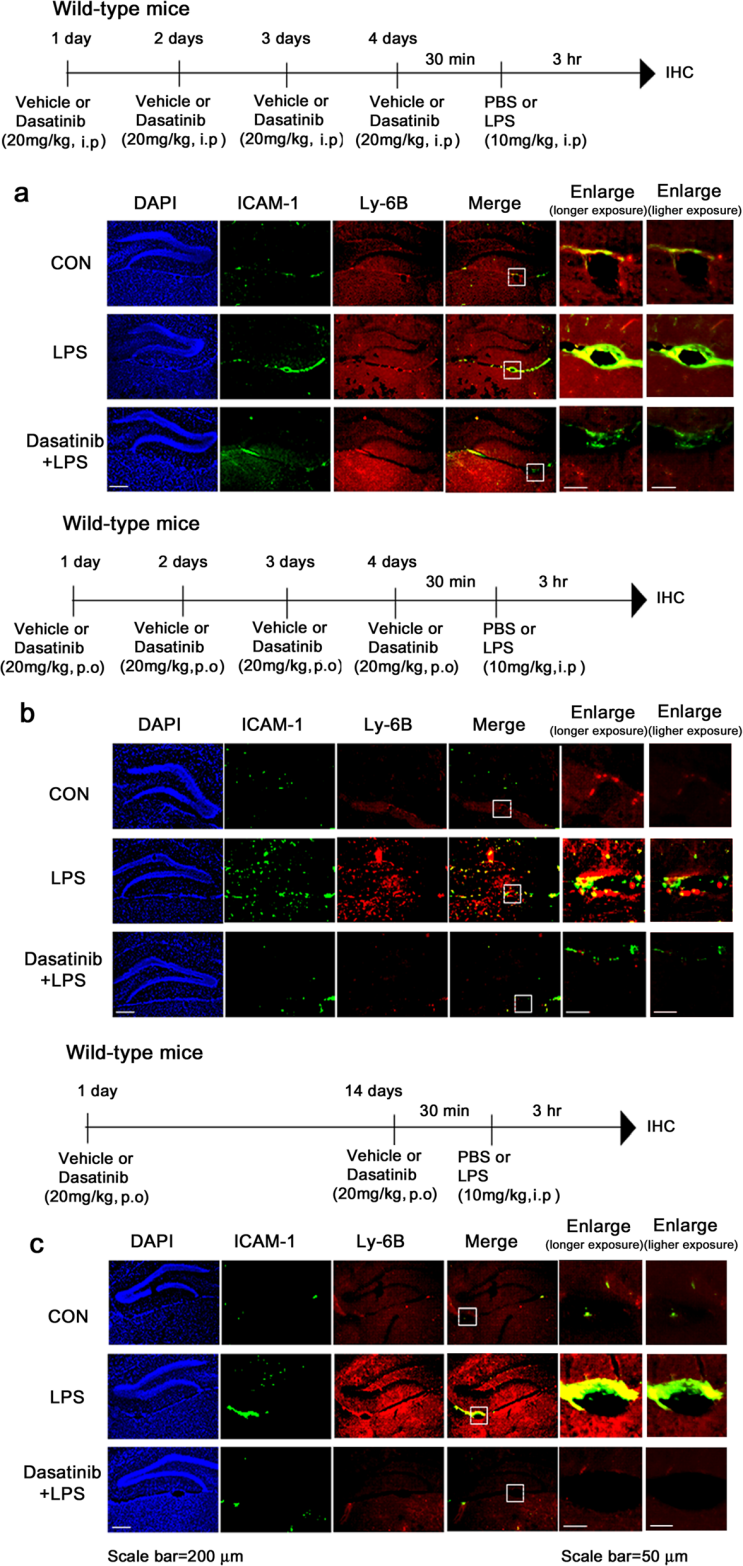
Dasatinib decreases LPS-stimulated ICAM-1-interacting neutrophil rolling in wild-type mice. **a** Wild-type mice were injected with dasatinib (20 mg/kg, i.p.) or vehicle (4% DMSO + 30% PEG + 5% Tween 80, i.p.) daily for 4 days, followed by injection with LPS (10 mg/kg, i.p.) or PBS. Three hours after LPS or PBS injection, immunohistochemistry was conducted with anti-ICAM-1 and Ly-6B antibodies. **b** Wild-type mice were orally administered dasatinib (20 mg/kg, p.o.) or vehicle (4% DMSO + 30% PEG + 5% Tween 80, i.p.) daily for 4 days, followed by injection with LPS (10 mg/kg, i.p.) or PBS. Three hours after LPS or PBS injection, immunohistochemistry was conducted with anti-ICAM-1 and Ly-6B antibodies. **c** Wild-type mice were orally administered dasatinib (20 mg/kg, p.o.) or vehicle (4% DMSO + 30% PEG + 5% Tween 80, i.p.) daily for 2 weeks, followed by injection with LPS (10 mg/kg, i.p.) or PBS. Three hours after LPS or PBS injection, immunohistochemistry was conducted with anti-ICAM-1 and Ly-6B antibodies

To examine whether dasatinib affects LPS-induced neutrophil rolling in the CNS vasculature, wild-type mice were injected with dasatinib (20 mg/kg, i.p. daily for 4 days, 20 mg/kg, p.o. daily for 4 days, or 20 mg/kg, p.o. daily for 2 weeks) or vehicle (4% DMSO + 30% PEG + 5% Tween 80), followed by injection with LPS (10 mg/kg, i.p.) or PBS. Three hours after LPS or PBS injection, the mice were perfused and fixed, and immunohistochemistry was performed with anti-Ly-6B (neutrophil marker) and anti-ICAM-1 (endothelial cell marker) antibodies. We observed increased neutrophil rolling in LPS-injected wild-type mice, whereas intraperitoneal injection of dasatinib exhibits a tendency toward decreased endothelial ICAM-1-interacting neutrophils in the CNS vasculature of LPS-injected wild-type mice (Fig. 16a). In parallel experiments, we orally administered dasatinib daily for 4 or 14 days and found that oral administration of dasatinib tendency to suppress endothelial ICAM-1-interacting neutrophils in the CNS vasculature of LPS-injected wild-type mice (Fig. 16b, c).

## Discussion

Our results demonstrate that the multi-target oral tyrosine kinase inhibitor dasatinib regulates LPS-induced microglial and astrocytic neuroinflammatory responses. In BV2 microglial cells, dasatinib inhibits TLR4/ERK or TLR4/AKT signaling to reduce LPS-induced proinflammatory cytokine COX-2 or IL-6 mRNA levels. In primary microglial and primary astrocytes, dasatinib modulates LPS-induced AKT or ERK signaling to alter LPS-stimulated proinflammatory cytokine levels. In addition, dasatinib-treated BV2 microglial cells and primary astrocytes suppress LPS-stimulated nuclear STAT3 phosphorylation. Moreover, dasatinib downregulates LPS-induced microglial/astrocyte activation, proinflammatory cytokine levels in the brain and plasma, and neutrophil rolling in LPS-treated wild-type mice. Collectively, our findings suggest that dasatinib can regulate LPS-induced neuroinflammatory responses in BV2 microglial cells, primary astrocytes, primary microglia, and wild-type mice.

In this study, we used LPS as a stimulator of inflammation to determine the effects of dasatinib on LPS-stimulated neuroinflammatory responses. Interestingly, several studies have found that the activity of the tyrosine kinase c-Abl increases LPS-induced inflammation in macrophages, BV2 microglial cells, and a mouse model of acute pancreatitis [23–25]. The Src tyrosine kinase family regulates local inflammation in colorectal cancer and LPS-induced inflammatory responses [26, 27]. Yoo et al. also found that the dopamine receptor agonist aripiprazole reduces proinflammatory cytokine levels by inhibiting Src kinase activity in macrophages [28]. These findings suggest that inhibition of these tyrosine kinases (including c-Abl and Src) may be a useful therapeutic approach to regulate inflammation. Indeed, several studies have demonstrated that the multi-target tyrosine kinase inhibitor dasatinib (which targets Bcr-Abl, c-Abl, Arg, Src, BTK, and Tec) can reduce pulmonary inflammation by decreasing M1 macrophages and increasing M2 macrophages [10, 11, 29]. In addition, Lawana et al. reported that pre-treatment with dasatinib (100 nM) significantly reduces LPS/rotenone-evoked iNOS levels in BV2 microglial cells [25]. Fraser et al. found that pre-treatment with dasatinib (500 nM) significantly reduces LPS-induced TNF-α levels in RAW 264.7 cells [12]. However, whether post-treatment with dasatinib can alter LPS-stimulated neuroinflammatory responses has not been studied in detail. More importantly, whether dasatinib modulates LPS-induced neuroinflammation in primary microglial and primary astrocytes and its mechanisms of action in BV2 microglial cells, primary microglial cells, and primary astrocytes remains unclear.

Under our experimental conditions, 250 nM dasatinib decreased LPS-induced proinflammatory cytokine levels in BV2 microglial cells more effectively than treatment with 100 nM dasatinib (Fig. 1, Additional file 1: Figure S2). In addition, post-treatment with dasatinib (as a curative condition) reduced LPS-evoked neuroinflammatory responses in BV2 microglial cells less effectively than pre-treatment with dasatinib (as a preventive condition) (Fig. 1, Additional file 1: Figure S4). Based on our findings, we further tested whether dasatinib differentially affects LPS-induced proinflammatory cytokine levels under low-glucose conditions, as high glucose levels can activate glial cells to produce inflammatory responses [30]. Interestingly, dasatinib treatment significantly reduced LPS-induced iNOS and TNF-α mRNA levels in primary microglial cells under low-glucose conditions (Fig. 2). By contrast, dasatinib treated primary microglial cells significantly downregulated LPS-induced COX-2 and TNF-α mRNA levels under high-glucose conditions (Fig. 2). In primary astrocytes, 250 nM dasatinib significantly suppressed LPS-induced proinflammatory cytokine COX-2 and iNOS mRNA levels under both low- and high-glucose conditions (Fig. 7e–p). Moreover, dasatinib regulated anti-inflammatory cytokine levels in LPS-induced BV2 microglial cells, primary microglial cells, and primary astrocytes (Fig. 2, Additional file 1: Figures S1 and S7). Collectively, these data suggest that pre- or post-treatment with dasatinib differentially affects LPS-induced proinflammatory cytokine levels depending on the cell type, glucose level (high vs low glucose), and experimental procedure (i.e., concentration of dasatinib, duration of treatment).

TLR4 is the predominant receptor by which LPS mediates immune activation [31, 32]. For instance, binding of LPS to TLR4 on the surface of microglia activates TLR4 and downstream signaling pathways [33]. Activated TLR4 signaling affects NF-κB/STAT3 and/or other transcription factors in the nucleus and triggers the release of proinflammatory cytokines [34]. We and others have demonstrated that several tyrosine kinase inhibitors regulate LPS-TLR4 signaling to modulate neuroinflammatory responses. For example, we previously reported that the Bruton’s tyrosine kinase (BTK) inhibitor ibrutinib inhibits TLR4 signaling and regulates LPS-induced neuroinflammatory responses in BV2 microglial cells [22]. Another BTK inhibitor, LFM-A13, prevents TLR4-dependent signaling in LPS-treated THP-1 human monocytic cells [35]. In addition, the Src kinase inhibitors PP1 and PP2 suppress LPS-TLR4 signaling in human embryonic kidney cells transfected with human TLR4 and MD-2 [36]. Thus, modulating TLR4 signaling is a potential therapeutic strategy for preventing/treating neuroinflammation-related diseases. In the present study, we found that dasatinib (whose targets include BTK and Src) downregulated LPS-induced COX-2 mRNA levels in a TLR4-dependent manner in BV2 microglial cells (Fig. 3). In addition, dasatinib reduced LPS-induced IL-6 mRNA levels in a manner partially dependent on TLR4 signaling in BV2 microglial cells (Fig. 3). These data suggest that dasatinib alters other neuroinflammatory-related signaling pathways to regulate LPS-induced proinflammatory cytokine IL-6 mRNA levels. Taken together, dasatinib affects TLR4 signaling to modulate LPS-induced proinflammatory cytokine levels.

**Fig. 17:**
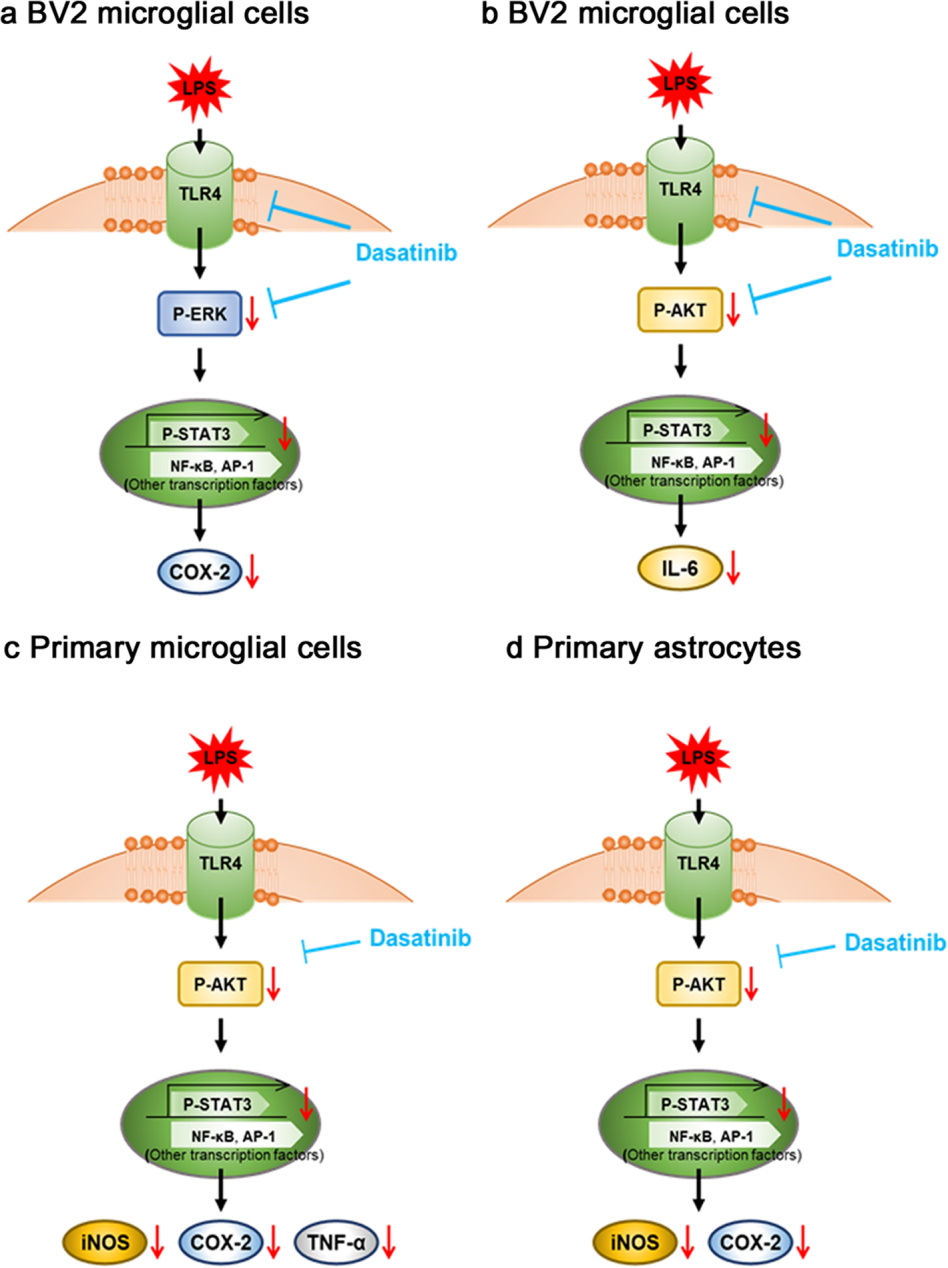
A working model for how dasatinib alters LPS-induced proinflammatory responses in BV2 microglial cells, primary microglial cells, and primary astrocytes. **a** In BV2 microglial cells, dasatinib treatment inhibits TLR4/ERK signaling, leading to suppression of LPS-induced nuclear p-STAT3 and/or other transcription factors and, in turn, reduced LPS-induced proinflammatory cytokine COX-2 mRNA levels. **b** Dasatinib treatment inhibits TLR4/AKT signaling in BV2 microglial cells, leading to suppressed LPS-induced proinflammatory cytokine IL-6 mRNA levels. **c** Dasatinib treatment of primary microglial cells inhibits AKT signaling and results in downregulation of LPS-induced proinflammatory cytokine levels. **d** In primary astrocytes, dasatinib treatment prevents AKT phosphorylation, which decreases LPS-induced nuclear p-STAT3 levels and subsequently leads to downregulation of LPS-induced proinflammatory cytokine levels

AKT and ERK signaling are associated with LPS-induced immune responses via TLR4 signaling [37]. For instance, knocking out AKT markedly reduces proinflammatory cytokine levels in human liver cancer cell lines [38]. Saponaro et al. found that AKT signaling promotes LPS-induced proinflammatory cytokine iNOS levels in microglial cells [39]. Signaling by another main kinase, ERK, is also involved in proinflammatory cytokine release [40]. Specifically, Seo et al. found that ERK inhibition downregulates LPS-induced NF-κB levels in mouse primary osteoblasts [40]. In addition, a recent study found that in myeloid cells (including monocytes, macrophage, microglial cells), TLR4/PI3K/AKT signaling alters proinflammatory cytokine IL-6 levels, whereas TLR4/TRAF6/ERK signaling regulates proinflammatory cytokine COX-2 levels [20]. Therefore, regulation of AKT and ERK signaling may play an important role in controlling inflammatory responses. Interestingly, we and others have observed associations between several tyrosine kinases (i.e., BTK, Src, Tec) and AKT and ERK signaling in inflammation- and neuroinflammation-associated diseases (i.e., cancer, CML). For instance, we recently demonstrated that the BTK inhibitor ibrutinib downregulates AKT phosphorylation to suppress proinflammatory cytokine COX-2 and IL-1β levels in BV2 microglial cells [22]. Herman et al. demonstrated that the selective BTK inhibitor acalabrutinib decreases ERK phosphorylation in mononuclear cells from a mouse model of chronic lymphocytic leukemia [41]. The Tec kinase inhibitor LFM-A13 suppresses LPS-evoked TNF-α, IL-6, and IL-1β levels but not P38 signaling via regulation of JNK phosphorylation in human neutrophils [42]. The combination of dasatinib with other anti-cancer drugs has been reported to prevent AKT and ERK signaling to suppress cancer cell migration or the proliferation of pancreatic and ovarian cancer cells [43, 44]. In addition, dasatinib alone exhibits anti-tumor efficacy by inhibiting ERK/AKT signaling in nasopharyngeal carcinoma cells [45]. However, whether dasatinib, a multi-target oral tyrosine kinase inhibitor, alters microglial and astrocytic LPS-induced AKT and ERK signaling to regulate LPS-stimulated proinflammatory cytokine levels has not been examined in detail. In the present study, we observed that dasatinib itself reduced AKT and/or ERK phosphorylation in BV2 microglia and primary astrocytes (Additional file 1: Figures S5 and S8). In addition, dasatinib significantly reduced LPS-induced AKT and/or ERK phosphorylation in BV2 microglial cells, primary microglial cell, and primary astrocytes (Fig. 4, Additional file 1: Figures S5 and S8). These results indicate that dasatinib can suppress the basal level of phosphorylation of AKT and ERK as well as the levels of phosphorylation evoked by LPS. Moreover, we observed that dasatinib treatment inhibited LPS-induced TLR4/AKT signaling to reduce LPS-stimulated IL-6 mRNA levels in BV2 microglial cells (Figs. 5a–c and 17). Dasatinib-treated BV2 microglial cells decreased LPS-induced TLR4/ERK signaling to suppress LPS-stimulated COX-2 mRNA levels (Figs. 5d–f and 17). Based on the literature and our findings, we suggest that dasatinib exerts anti-inflammatory effects by regulating AKT and/or ERK signaling pathways to modulate LPS-induced proinflammatory cytokine levels in BV2 microglial cells, primary microglial cells, and primary astrocytes. Future studies will investigate the molecular mechanism by which dasatinib works through the AKT and/or ERK signaling pathway to reduce the levels of individual LPS-stimulated proinflammatory cytokines using shRNA (i.e., TLR4, ERK, AKT) and/or knock-out mice.

LPS promotes levels of the transcription factors NF-kB and/or STAT3 in the nucleus to induce proinflammatory cytokine production [46–49]. Several recent studies have found that tyrosine kinases and/or tyrosine kinase inhibitors can regulate LPS-induced NF-kB and/or STAT3 levels in the nucleus to alter neuroinflammation. For instance, the tyrosine kinase Bcr-Abl stimulates STAT3 (Ser727) phosphorylation via JAK/MEK signaling, whereas the Bcr-Abl kinase inhibitor ponatinib suppresses IL-6-stimulated STAT3 phosphorylation in human colorectal cancer (CRC) cells [50, 51]. We also previously reported that the BTK inhibitor ibrutinib regulates LPS-induced neuroinflammation by suppressing STAT3 signaling in BV2 microglial cells [22]. Pre-treatment with the Src inhibitor PP1 suppresses LPS-induced NF-κB signaling in mouse lung tissue and mouse alveolar macrophages [52]. Tan et al. demonstrated that dasatinib and JAK inhibitors (i.e., ruxolitinib and tofacitinib) exhibit anti-STAT3 efficacy in human CRC cells [51]. Another study showed that dasatinib suppresses the phosphorylation of STAT3/STAT5 in leukemia-initiating cells [53]. Here, we observed that the multi-target oral tyrosine kinase inhibitor dasatinib significantly downregulated LPS-stimulated cytosolic and nuclear STAT3 phosphorylation in BV2 microglial cells and primary astrocytes (Figs. 6 and 8). Surprisingly, we observed that the effects of dasatinib on LPS-induced proinflammatory cytokine levels were partially dependent on STAT3 signaling. It is possible that dasatinib affects other transcription factors (e.g., NF-kB, AP-1, and STAT5) and/or unknown transcription factors to further modulate LPS-stimulated proinflammatory cytokine levels. A second possibility is that dasatinib influences AKT/ERK phosphorylation and/or other neuroinflammation-associated signaling (i.e., JAK/MEK) to synergistically affect LPS-induced proinflammatory cytokine production. Future studies will investigate whether dasatinib modulates known/unknown transcription factors to affect the levels of individual LPS-stimulated proinflammatory cytokines.

Several studies have examined whether tyrosine kinases and/or tyrosine kinase inhibitors can regulate LPS-induced inflammation in the brain and/or other organs/cells. For instance, LPS-induced pAKT/pNFκB/iNOS levels in bone marrow-derived macrophages (BMDMs) are significantly reduced in BTK knockdown mice [54]. Ni Gabhann et al. found that the BTK inhibitor LFM-A13 suppresses the LPS-mediated increase in IL-1β levels in wild-type murine peritoneal macrophages [55]. We previously reported that the BTK inhibitor ibrutinib significantly decreases LPS-induced microglial/astrocytic activation and proinflammatory cytokine COX-2 and IL-1β levels in wild-type mice [22]. Other tyrosine kinase BCR-ABL inhibitors (i.e., imatinib and nilotinib) suppress LPS-stimulated TNF-α, IL-6, and IL-1β levels in bronchoalveolar lavage fluid in a lung injury mouse model [56]. Interestingly, a recent study demonstrated that the multi-target tyrosine kinase inhibitor dasatinib (at doses of 5 to 50 mg/kg) inhibits the increase in serum TNF-α induced by 20 mg/kg LPS in wild-type mice [12].

Here, we further investigated whether dasatinib can modulate LPS-induced neuroinflammatory responses in the brain and found that intraperitoneal and oral administration of dasatinib (20 mg/kg, i.p. or p.o.) decreased LPS-evoked microglial/astrocytic activation as well as proinflammatory cytokine levels in LPS-injected wild-type mice (Figs. 9, 10, 11, 12, 13 and 14). In addition, we examined the effect of dasatinib on peripheral inflammatory responses and found that intraperitoneal and oral administration of dasatinib (20 mg/kg, i.p. or p.o.) reduced the LPS-induced plasma proinflammatory cytokine levels in LPS-injected wild-type mice (Fig. 15). These data suggest that dasatinib can regulate LPS-induced microglial/astrocyte activation and proinflammatory cytokine levels in the brain and plasma.

Intracerebral LPS injection was reported to significantly increase neutrophil infiltration in the hippocampus in ME7-prion diseased mice [57]. In addition, systemic administration of LPS increases TLR4-dependent leukocyte rolling and adhesion in the brain vessels of wild-type mice [58]. Endothelial ICAM-1 is essential for leukocyte rolling in the inflammatory response [59], systemic LPS injection significantly increases ICAM-1 expression in wild-type mice, and LPS-recruited neutrophils adhere to ICAM-1-positive CNS vasculature [60]. In this study, we found that intraperitoneal and oral administration of dasatinib regulated LPS-induced neutrophil rolling in the CNS vasculature (Fig. 16), suggesting that dasatinib can modulate LPS-induced central and peripheral inflammation.

Dasatinib is known to have effective treatment in several types of cancer, including CML. For instance, a recent study demonstrated that the combination of dasatinib and quercetin (pentahydroxyflavone with chemopreventive activity) has senolytic effects by reducing proinflammatory cytokine levels in senescent cell-transplanted young-aged mice [61]. Azizi et al. reported that dasatinib exhibited therapeutic effects in a mouse experimental autoimmune encephalomyelitis (EAE) model by decreasing proinflammatory cytokine NO production and TNF-α levels, and Oliveira et al. found that dasatinib inhibits LPS-induced lung inflammation in mice [62, 63]. In addition, Askmyr et al. observed an inflammatory response in a mouse model of CML based on increase in the numbers of macrophages and T-cells [64]. A couple of studies have reported that a proportion of patients with CML treated with imatinib, a therapeutic drug for CML, leading to the development of CNS leukemia [65–67]. These studies revealed that imatinib does not effectively cross the blood-brain barrier (BBB) into the CNS. As a result, it is difficult to inhibit CML in the CNS, leading to the development of CNS leukemia. By contrast, dasatinib has been reported to cross the BBB and to show higher treatment effects in CML patients compared with imatinib [68, 69]. However, the effects of dasatinib on neuroinflammation in the brain are not well studied.

Emerging evidence has implicated neuroinflammation as a major mechanism of neurodegenerative diseases, including AD [70–78]. Excessive activation of microglia and astrocytes results in the secretion of proinflammatory mediators, leading to neuroinflammation. In addition, enhanced glia-mediated neuroinflammatory responses and increased levels of pro-inflammatory mediators in the brain have been observed in an AD animal model and AD patients [70–78]. Therefore, regulation of primary astrocyte/microglial cell-mediated neuroinflammation is a potential therapeutic target to treat neurodegenerative diseases. In this study, we revealed that intraperitoneal and oral administration of dasatinib modulated LPS-induced neuroinflammation in LPS-treated wild-type mice (Figs. 9, 10, 11, 12, 13, and 14). Taken together, our results suggest that dasatinib can modulate glia-induced neuroinflammatory responses and anti-inflammatory effects in the brain and may be an effective treatment for CNS leukemia, neuroinflammation-related diseases, and neurodegenerative diseases (i.e., AD).

## Conclusions

Dasatinib regulates LPS-induced proinflammatory cytokine levels in BV2 microglial cells, primary microglial cells, and primary astrocytes and modulates TLR4 signaling to inhibit LPS-induced neuroinflammatory responses. In addition, dasatinib affects LPS-induced downstream AKT and/or ERK signaling to alter neuroinflammation in BV2 microglial cells, primary microglial cells, and primary astrocytes (Fig. 17). Moreover, intraperitoneal and oral administration of dasatinib significantly reduces LPS-induced microglial/astrocyte activation, proinflammatory cytokine levels in the brain and plasma, and neutrophil rolling in the brain. Taken together, our results suggest that dasatinib affects LPS-induced microglial and astrocytic neuroinflammation through AKT/STAT3 signaling.

## Additional file

Additional file 1: **Figure S1.** Treatment with 100 nM dasatinib significantly reduces LPS-induced proinflammatory cytokine COX-2 and IL-6 mRNA levels in BV2 microglial cells. **Figure S2.** Treatment with 250 nM dasatinib significantly reduces low-dose LPS-induced proinflammatory cytokine COX-2, IL-6, and TNF-α mRNA levels in BV2 microglial cells. **Figure S3.** Treatment with 250 nM dasatinib regulates anti-inflammatory cytokine *Il4* and *Il10* levels in BV2 microglial cells in the presence of LPS. **Figure S4.** Post-treatment with dasatinib significantly reduces LPS-induced proinflammatory cytokine COX-2 mRNA levels. **Figure S5.** Dasatinib does not alter LPS-induced p-P38 levels in BV2 microglial cells. **Figure S6.** Dasatinib significantly inhibits 200 ng/ml LPS-induced AKT/ERK phosphorylation. **Figure S7.** Treatment with dasatinib increases anti-inflammatory cytokine *Il10* levels in mouse primary astrocytes in the presence of LPS. **Figure S8.** Dasatinib treatment does not reduce LPS-induced p-P38 levels in mouse primary astrocytes. (DOCX 1020 kb)

## Data Availability

All data generated and/or analyzed during this study are included in this article.

## Abbreviations

AD: Alzheimer’s disease
BTK: Bruton’s tyrosine kinase
CML: Chronic myeloid leukemia
COX-2: Cyclooxygenase-2
IL-1β: Interleukin-1β
IL-6: Interleukin-6
LPS: Lipopolysaccharide
STAT3: Signal transducer and activator of transcription 3
TLR4: Toll-like receptor 4
TNF-α: Tumor necrosis factor-α
