# Supplementary material for: Dasatinib regulates LPS-induced microglial and astrocytic neuroinflammatory responses by inhibiting AKT/STAT3 signaling
Source: J Neuroinflammation. 2019 Oct 26;16:190. doi: 10.1186/s12974-019-1561-x (PMC6815018; doi:10.1186/s12974-019-1561-x)
Supplement: Supplementary file 1 — Figure S1. Treatment with 100 nM dasatinib significantly reduces LPS-induced proinflammatory cytokine COX-2 and IL-6 mRNA levels in BV2 microglial cells. Figure S2. Treatment with 250 nM dasatinib significantly reduces low-dose LPS-induced proinflammatory cytokine COX-2, IL-6, and TNF-α mRNA levels in BV2 microglial cells. Figure S3. Treatment with 250 nM dasatinib regulates anti-inflammatory cytokine Il4 and Il10 levels in BV2 microglial cells in the presence of LPS. Figure S4. Post-treatment with dasatinib significantly reduces LPS-induced proinflammatory cytokine COX-2 mRNA levels. Figure S5. Dasatinib does not alter LPS-induced p-P38 levels in BV2 microglial cells. Figure S6. Dasatinib significantly inhibits 200 ng/ml LPS-induced AKT/ERK phosphorylation. Figure S7. Treatment with dasatinib increases anti-inflammatory cytokine Il10 levels in mouse primary astrocytes in the presence of LPS. Figure S8. Dasatinib treatment does not reduce LPS-induced p-P38 levels in mouse primary astrocytes. (DOCX 1020 kb) [file 12974_2019_1561_MOESM1_ESM.docx]

**Dasatinib regulates LPS-induced microglial and astrocytic neuroinflammatory responses by inhibiting AKT/STAT3 signaling**

Ka-Young Ryu^1,3^, Hyun-ju Lee^1,3^, Hanwoong Woo^1^, Ri-Jin Kang^1^, Kyung-Min Han^1,2^, HyunHee Park^1^, Sang Min Lee^1^, Ju-Young Lee^1^, Yoo Joo Jeong^1^, Hyun-Wook Nam^1^, Youngpyo Nam^1,*^, Hyang-Sook Hoe^1,2,*^

^1^Department of Neural Development and Disease, Korea Brain Research Institute (KBRI), 61, Cheomdan-ro, Dong-gu, Daegu, Korea 41068; ^2^Department of Brain & Cognitive Sciences, Daegu Gyeongbuk Institute of Science & Technology (DGIST), 333 Techno Jungang-daero, Hyeonpung-myeon, Dalseong-gun, Daegu, Korea, 42988; ^3^These authors contributed equally to this study.

Ka-Young Ryu: rkfud1208@kbri.re.kr

Hyun-ju Lee: hjlee@kbri.re.kr

Hanwoong Woo: hwwoo@kbri.re.kr

Ri-Jin Kang: flwls2001@kbri.re.kr

Kyung-Min Han: hkm5344@gmail.com

HyunHee Park: [hyunhee16hh@gmail.com](mailto:hyunhee16hh@gmail.com)

Sang Min Lee: lsm6897@kbri.re.kr

Ju-Young Lee: [dear678@naver.com](mailto:dear678@naver.com)

Yoo Joo Jeong: yoojoo930@gmail.com

Hyun-Wook Nam: nhw0121@gmail.com

Youngpyo Nam: ypnam@kbri.re.kr

Hyang-Sook Hoe: [sookhoe72@kbri.re.kr](mailto:sookhoe72@kbri.re.kr)

^*^Corresponding author

Hyang-Sook Hoe, Ph.D., Department of Neural Development and Disease, Korea Brain Research Institute (KBRI), 61, Cheomdan-ro, Dong-gu, Daegu, Korea 41068

E-mail: [sookhoe72@kbri.re.kr](mailto:sookhoe72@kbri.re.kr)

Youngpyo Nam, Ph.D., Department of Neural Development and Disease, Korea Brain Research Institute (KBRI), 61, Cheomdan-ro, Dong-gu, Daegu, Korea 41068

E-mail: [ypnam@kbri.re.kr](mailto:ypnam@kbri.re.kr)


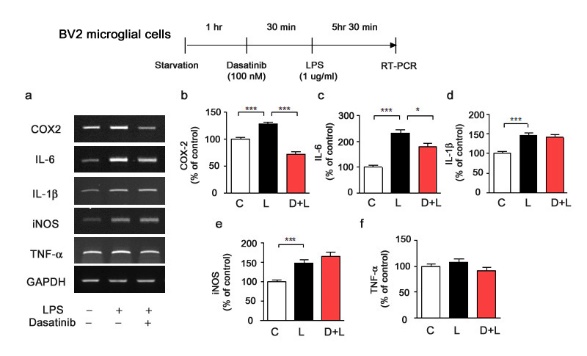


**Figure S1** Treatment with 100 nM dasatinib significantly reduces LPS-induced proinflammatory cytokine COX-2 and IL-6 mRNA levels in BV2 microglial cells. **a** BV2 microglial cells were treated with dasatinib (100 nM) or vehicle (1% DMSO) for 30 min followed by LPS (1 μg/ml) or PBS for 5.5 hr, and proinflammatory cytokine levels were analyzed. **b-f** Quantification of the data in **a** (COX-2, IL-6, IL-1β, iNOS, and TNF-α: con, n = 24; LPS, n = 24; Dasatinib+LPS, n = 24). One-way ANOVA with Tukey’s post hoc test was used to analyze significant differences. *p < 0.05, ***p < 0.001


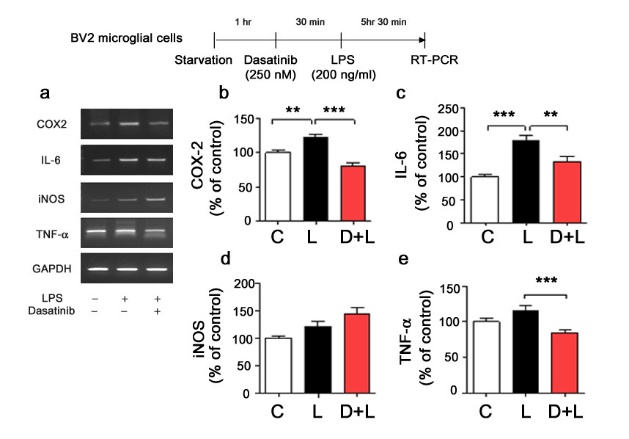


**Figure S2** Treatment with 250 nM dasatinib significantly reduces low-dose LPS-induced proinflammatory cytokine COX-2, IL-6, and TNF-α mRNA levels in BV2 microglial cells. **a** BV2 microglial cells were treated with dasatinib (250 nM) or vehicle (1% DMSO) for 30 min followed by LPS (200 ng/ml) or PBS for 5.5 hr, and proinflammatory cytokine levels were analyzed. **b-e** Quantification of the data in **a** (COX-2, IL-6, iNOS, and TNF-α: con, n = 18; LPS, n = 18; dasatinib+LPS, n = 18). One-way ANOVA with Tukey’s post hoc test was used to analyze significant differences. **p < 0.01, ***p < 0.001


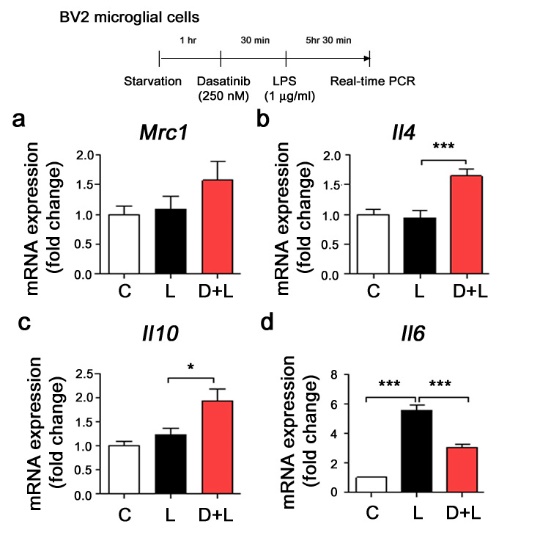


**Figure S3** Treatment with 250 nM dasatinib regulates anti-inflammatory cytokine *Il4* and *Il10* levels in BV2 microglial cells in the presence of LPS. BV2 microglial cells were treated with dasatinib (250 nM) or vehicle (1% DMSO) for 30 min followed by LPS (1 μg/ml) or PBS for 5.5 hr, and the levels of the anti-inflammatory cytokines *Mrc1* (**a**), *Il4* (**b**), *Il10* (**c**) and the levels of the proinflammatory cytokine *Il6* (**d**) were measured using real-time PCR (n = 8/group). One-way ANOVA with Tukey’s post hoc test was used to analyze significant differences. *p < 0.05, ***p < 0.001


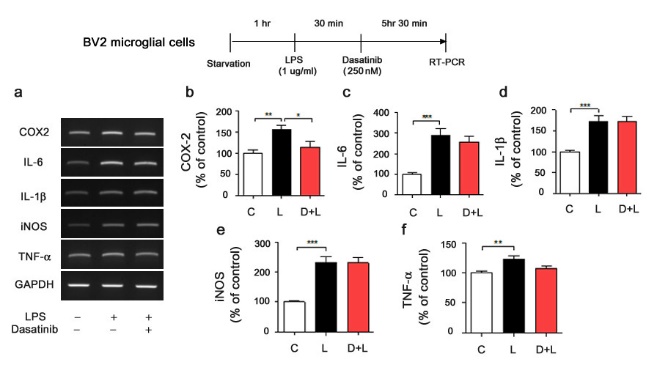


**Figure S4** Post-treatment with dasatinib significantly reduces LPS-induced proinflammatory cytokine COX-2 mRNA levels. **a** BV2 microglial cells were treated with LPS (1 μg/ml) or PBS for 30 min followed by dasatinib (250 nM) or vehicle (1% DMSO) for 5.5 hr, and proinflammatory cytokine levels were analyzed. **b-f** Quantification of the data in **a** (COX-2, IL-6, IL-1β, iNOS, and TNF-α: con, n = 18; LPS, n = 18; dasatinib+LPS, n = 18). One-way ANOVA with Tukey’s post hoc test was used to analyze significant differences. *p < 0.05, **p < 0.01, ***p < 0.001


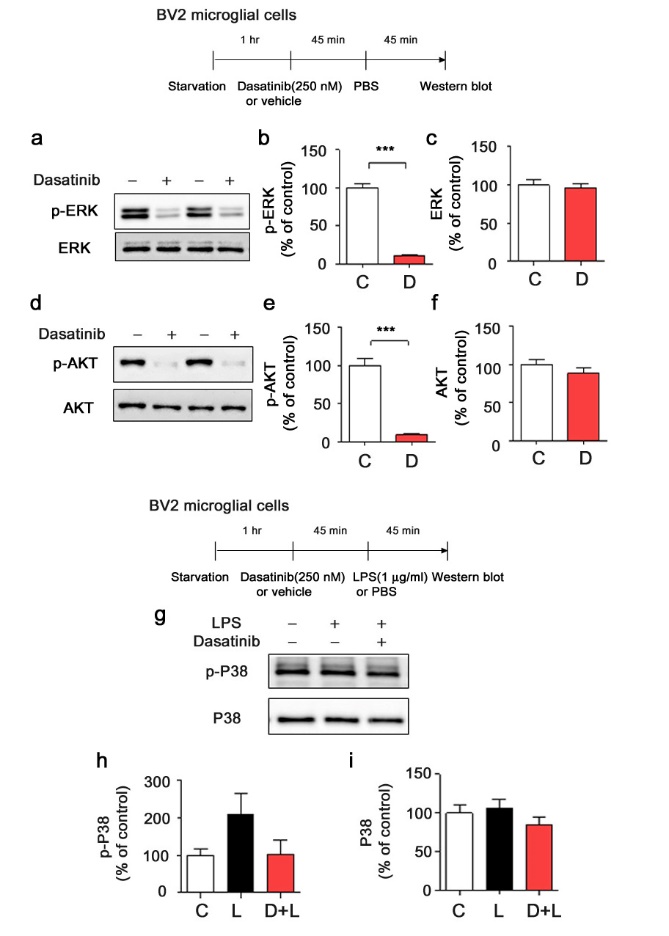


**Figure S5** Dasatinib does not alter LPS-induced p-P38 levels in BV2 microglial cells. **a** BV2 microglial cells were treated with dasatinib (250 nM) or vehicle (1% DMSO) for 45 min followed by PBS for 45 min, and western blotting was conducted with anti-p-ERK or anti-ERK antibodies. **b**-**c** Quantification of the data from **a** (p-ERK and ERK: con, n = 14; dasatinib, n = 14). **d** BV2 microglial cells were treated with dasatinib (250 nM) or vehicle (1% DMSO) for 45 min followed by PBS for 45 min, and western blotting was conducted with anti-p-AKT or anti-AKT antibodies. **e**-**f** Quantification of the data from **d** (p-AKT and AKT: con, n = 14; dasatinib, n = 14). **g** BV2 microglial cells were treated with dasatinib (250 nM) or vehicle (1% DMSO) for 45 min followed by LPS (1 μg/ml) or PBS for 45 min, and western blotting was conducted with anti-p-P38 or anti-P38 antibodies. **h-i** Quantification of the data in **g** (p-P38 and P38: con, n = 9; LPS, n = 9; dasatinib+LPS, n = 9). Two-tailed t-tests (**b-f**) and one-way ANOVA with Tukey’s post hoc test (**h-i**) were used to analyze significant differences ***p < 0.001


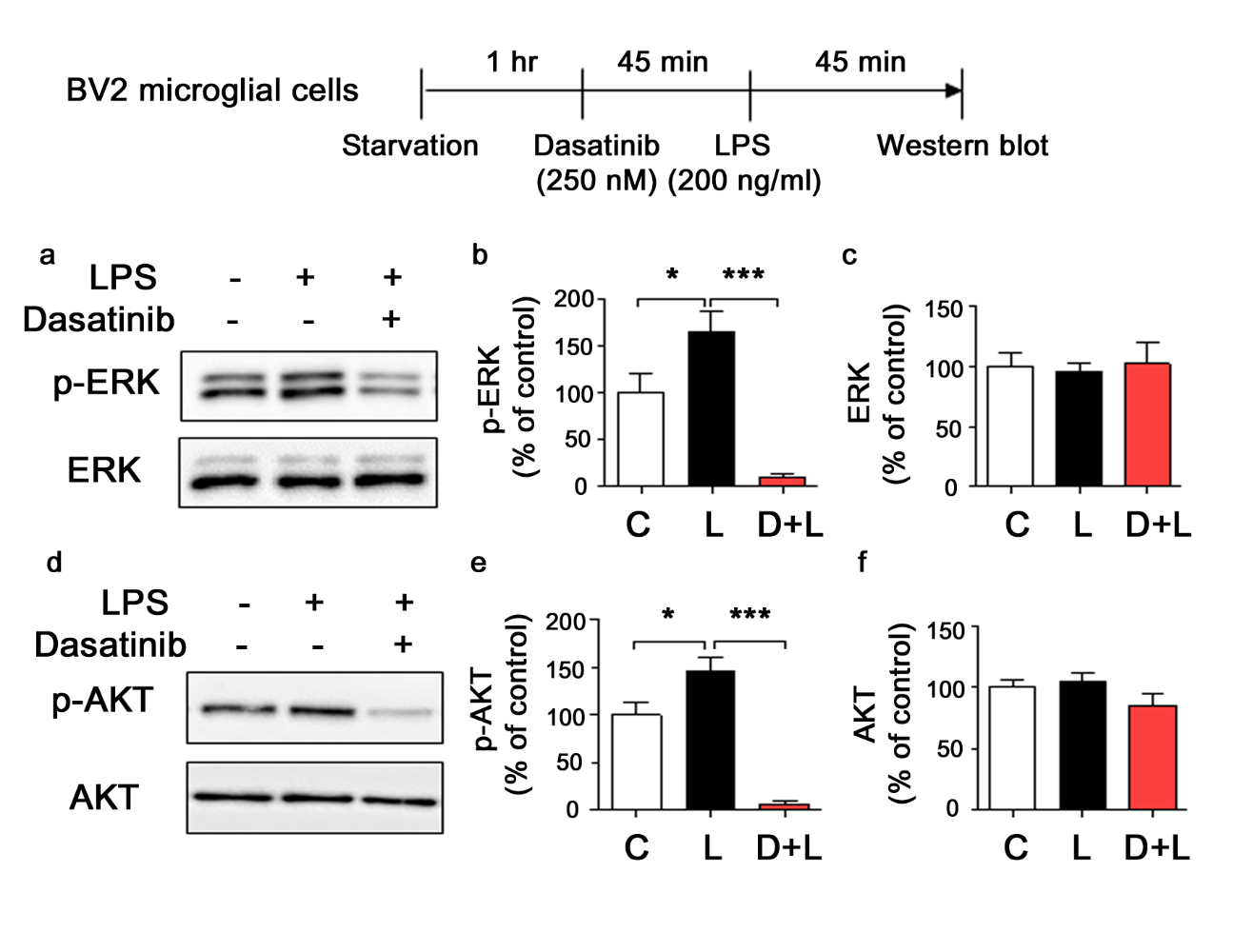


**Figure S6** Dasatinib significantly inhibits 200 ng/ml LPS-induced AKT/ERK phosphorylation. **a** BV2 microglial cells were treated with vehicle (1% DMSO) or dasatinib (250 nM) for 45 min followed by PBS or LPS (200 ng/ml) for 45 min, and western blotting was performed with anti-p-ERK or anti-ERK antibodies. **b–c** Quantification of the data from **a (**p-ERK and ERK: con, n = 6; LPS, n = 6; dasatinib+LPS, n = 6). **d** BV2 microglial cells were treated with vehicle (1% DMSO) or dasatinib (250 nM) for 45 min followed by PBS or LPS (200 μg/ml) for 45 min, and western blotting was conducted with anti-p-AKT or anti-AKT antibodies. **e–f** Quantification of the data from **d** (p-AKT and AKT: con, n = 6; LPS, n = 6; dasatinib+LPS, n = 6). One-way ANOVA with Tukey’s post hoc test was used to analyze significant differences. *p < 0.05, ***p < 0.001


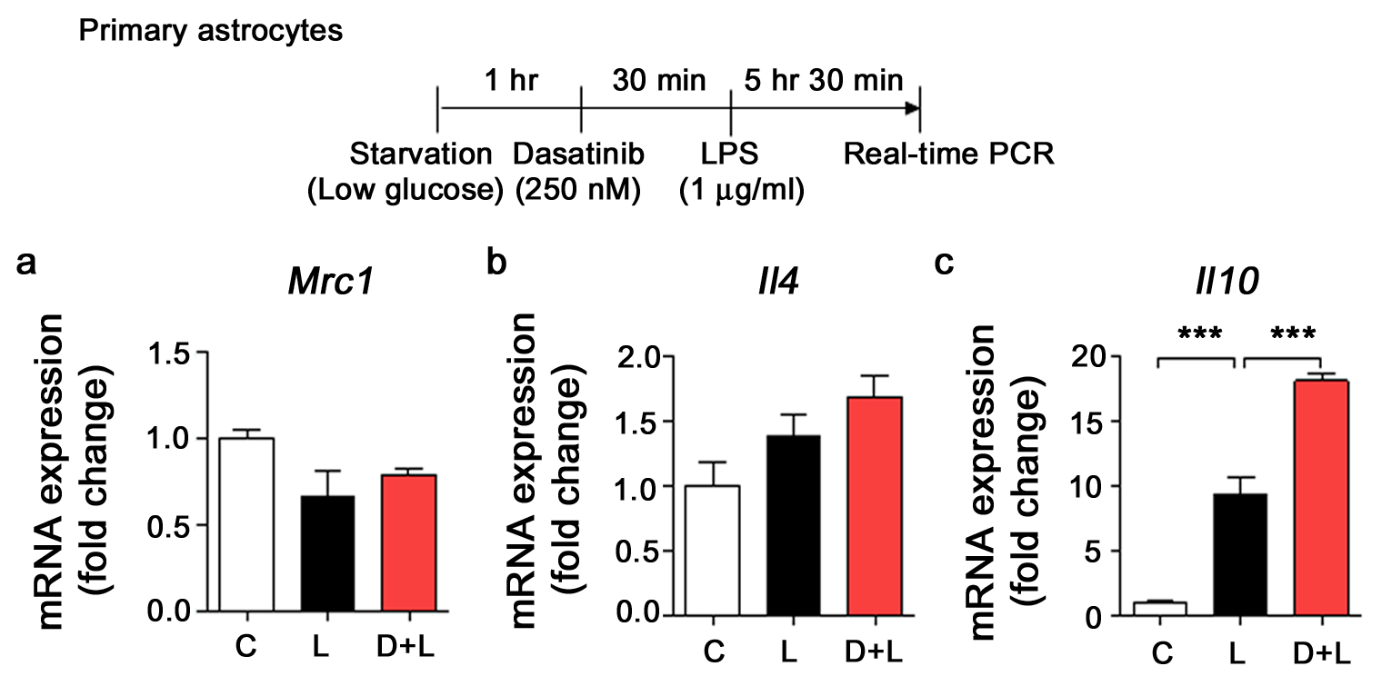


**Figure S7** Treatment with dasatinib increases anti-inflammatory cytokine *Il10* levels in mouse primary astrocytes in the presence of LPS. Primary astrocytes were treated with dasatinib (250 nM) or vehicle (1% DMSO) for 30 min followed by LPS (1 μg/ml) or PBS for 5.5 hr, and anti-inflammatory cytokine *Mrc1* (**a**), *Il4* (**b**), and *Il10* (**c**) levels were measured using real-time PCR (n = 4/group). One-way ANOVA with Tukey’s post hoc test was used to analyze significant differences. ***p < 0.001


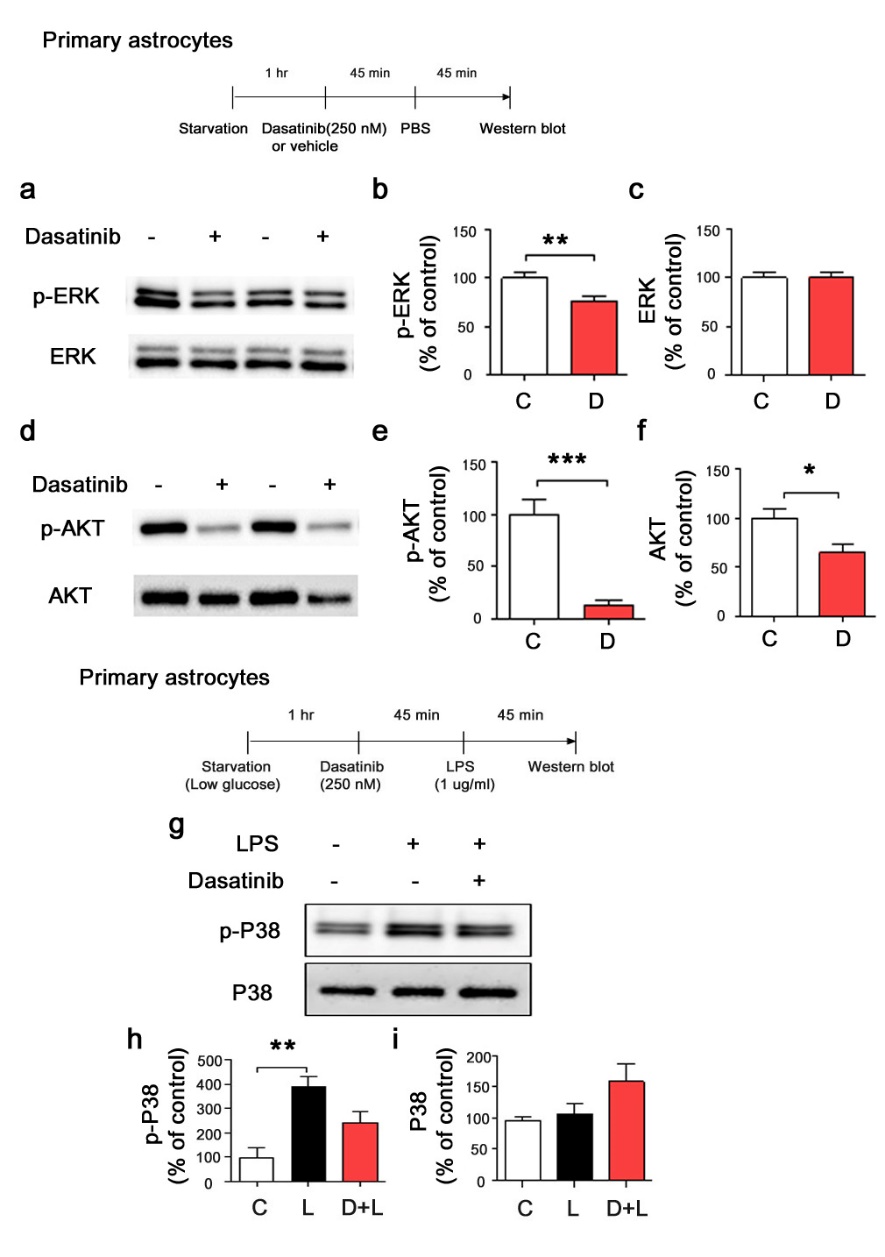


**Figure S8** Dasatinib treatment does not reduce LPS-induced p-P38 levels in mouse primary astrocytes. **a** Primary astrocytes were treated with dasatinib (250 nM) or vehicle (1% DMSO) for 45 min followed by PBS for 45 min, and western blotting was conducted with anti-p-ERK or anti-ERK antibodies. **b**-**c** Quantification of the data from **a** (p-ERK and ERK: con, n = 8; dasatinib, n = 8). **d** Primary astrocytes were treated with dasatinib (250 nM) or vehicle (1% DMSO) for 45 min followed by PBS for 45 min, and western blotting was conducted with anti-p-AKT or anti-AKT antibodies. **e**-**f** Quantification of the data from **d** (p-AKT and AKT: con, n = 8; dasatinib, n = 8). **g** Primary astrocytes were treated with dasatinib (250 nM) or vehicle (1% DMSO) for 45 min followed by LPS (1 μg/ml) or PBS for 45 min, and western blotting was conducted with anti-p-P38 or anti-P38 antibodies. **h-i** Quantification of the data in **g** (p-P38: con, n = 5; LPS, n = 5; dasatinib+LPS, n = 5; P38 con, n = 5; LPS, n = 5; dasatinib+LPS, n = 5). Two-tailed t-tests (**b-f**) and one-way ANOVA with Tukey’s post hoc test (**h-i**) were used to analyze significant differences **p < 0.01, ***p < 0.001
